# Length-of-Stay in the Emergency Department and In-Hospital Mortality: A Systematic Review and Meta-Analysis

**DOI:** 10.3390/jcm12010032

**Published:** 2022-12-21

**Authors:** Dominique Lauque, Anna Khalemsky, Zoubir Boudi, Linda Östlundh, Chang Xu, Mohammed Alsabri, Churchill Onyeji, Jacqueline Cellini, Geroge Intas, Kapil Dev Soni, Detajin Junhasavasdikul, Jose Javier Trujillano Cabello, Niels K. Rathlev, Shan W. Liu, Carlos A. Camargo, Anna Slagman, Michael Christ, Adam J. Singer, Charles-Henri Houze-Cerfon, Elhadi H. Aburawi, Karim Tazarourte, Lisa Kurland, Phillip D. Levy, James H. Paxton, Dionyssios Tsilimingras, Vijaya Arun Kumar, David G. Schwartz, Eddy Lang, David W. Bates, Gabriele Savioli, Shamai A. Grossman, Abdelouahab Bellou

**Affiliations:** 1 Department of Emergency Medicine, Purpan Hospital and Toulouse III University, 31300 Toulouse, France; 2 Department of Emergency of Medicine, Beth Israel Deaconess Medical Center, Teaching Hospital of Harvard Medical School, Boston, MA 02115, USA; 3 Management Department, Hadassah Academic College, Jerusalem 91010, Israel; 4 Department of Emergency Medicine, Dr Sulaiman Alhabib Hospital, Dubai 2542, United Arab Emirates; 5 Global Network on Emergency Medicine, Brookline, MA 02446, USA; 6 Örebro University Library, Örebro University, 70182 Örebro, Sweden; 7 Ministry of Education, Key Laboratory for Population Health Across-Life Cycle, Anhui Medical University, Hefei 230032, China; 8 Department of Pediatrics, Brookdale University Hospital and Medical Center, 1 Brookdale Plaza, Brooklyn, NY 11212, USA; 9 Countway Library, Harvard Medical School, Boston, MA 02115, USA; 10 MATHEMATICA, Inc., Princeton, NJ 08540, USA; 11 Department of Critical Care, General Hospital of Nikaia Agios Panteleimon, 18454 Athens, Greece; 12 Jai Prakash Narayan Apex Trauma Center, Ring Road, New Delhi 110029, India; 13 Department of Medicine, Faculty of Medicine Ramathibodi Hospital, Mahidol University, Bangkok 10400, Thailand; 14 Intensive Care Unit, Hospital Universitari Arnau de Vilanova, 25198 Lleida, Spain; 15 Department of Emergency Medicine, University of Massachusetts Medical School, Baystate, Springfield, MA 01199, USA; 16 Department of Emergency Medicine, Massachusetts General Hospital, Harvard Medical School, Boston, MA 02114, USA; 17 Division of Emergency and Acute Medicine, Campus Virchow Klinikum and Charité Campus Mitte, Charité Universitätsmedizin, 10117 Berlin, Germany; 18 Department of Emergency Medicine, 6000 Lucerne, Switzerland; 19 Department of Emergency Medicine, Renaissance Scholl of Medicine at Stony Brook University, Stony Brook, NY 11794, USA; 20 Department of Pediatrics, College of Medicine and Health Sciences, UAE University, Al Ain P.O. BOX 15551, United Arab Emirates; 21 Department of Health Quality, University Hospital, Hospices Civils, 69002 Lyon, France; 22 Department of Emergency Medicine, University Hospital, Hospices Civils, 69002 Lyon, France; 23 Department of Medical Sciences, Örebro University, 70182 Örebro, Sweden; 24 Department of Emergency Medicine, Wayne State University School of Medicine, Detroit, MI 48201, USA; 25 Department of Family Medicine & Public Health Sciences, Wayne State University School of Medicine, Detroit, MI 48201, USA; 26 Information Systems Department, Graduate School of Business Administration, Bar-Ilan University, Ramat-Gan 529002, Israel; 27 Department of Emergency Medicine, Emergency Medicine Cumming School of Medicine, University of Calgary, Alberta Health Services, Calgary, AB T2N 1N4, Canada; 28 Department of Healthcare Quality, Brigham and Women Hospital, Harvard Medical School, Boston, MA 02115, USA; 29 Emergency Department, IRCCS Fondazione Policlinico San Matteo, 27100 Pavia, Italy; 30 Institute of Sciences in Emergency Medicine, Department of Emergency Medicine, Guangdong Provincial People’s Hospital, Guangdong Academy of Medical Sciences, Guangzhou 510080, China

**Keywords:** emergency department, in-hospital mortality, intensive care unit, length-of-stay, meta-analysis, systematic review

## Abstract

The effect of emergency department (ED) length of stay (EDLOS) on in-hospital mortality (IHM) remains unclear. The aim of this systematic review and meta-analysis was to determine the association between EDLOS and IHM. We searched the PubMed, Medline, Embase, Web of Science, Cochrane Controlled Register of Trials, CINAHL, PsycInfo, and Scopus databases from their inception until 14–15 January 2022. We included studies reporting the association between EDLOS and IHM. A total of 11,337 references were identified, and 52 studies (total of 1,718,518 ED patients) were included in the systematic review and 33 in the meta-analysis. A statistically significant association between EDLOS and IHM was observed for EDLOS over 24 h in patients admitted to an intensive care unit (ICU) (OR = 1.396, 95% confidence interval [CI]: 1.147 to 1.701; *p* < 0.001, I^2^ = 0%) and for low EDLOS in non-ICU-admitted patients (OR = 0.583, 95% CI: 0.453 to 0.745; *p* < 0.001, I^2^ = 0%). No associations were detected for the other cut-offs. Our findings suggest that there is an association between IHM low EDLOS and EDLOS exceeding 24 h and IHM. Long stays in the ED should not be allowed and special attention should be given to patients admitted after a short stay in the ED.

## 1. Introduction

**Figure 1:**
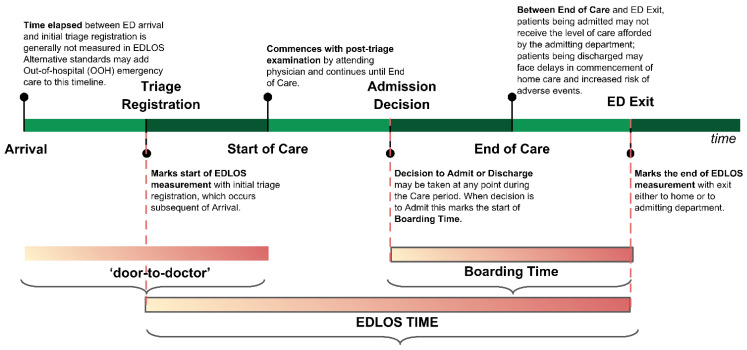
Definition of time spent in the emergency department related to each segment of the care process.

Prolonged length of stay (LOS) in the emergency department (ED), characterized by an inappropriately long period before final departure for an in-hospital bed, home, or another facility, is believed to adversely affect clinical outcomes. The time spent in the ED can be divided into distinct periods that are marked by time of arrival (triage registration), time of the start of care, time of the disposition decision (discharge or admission), time at the end of care, and time at ED departure (Figure 1). EDLOS is defined as the time elapsed between the initial triage registration and physical departure from the ED. Boarding time (BT), defined as the time spent waiting for inpatient bed availability after the decision to admit the patient is made, is a significant contributor to the LOS. BT may also affect outcomes, as boarded patients require ongoing, often intensive care that several EDs are not well equipped to deliver [1,2,3]. The definition of prolonged EDLOS may vary. Prolonged ED visits have been defined as >4 h in the United Kingdom, >6 h in Canada and the U.S., and >8 h in Australia [4,5,6].

Prior studies have shown that ED boarding delays care, including the commencement of home medication, and increases the risk of adverse events, prolongs in-hospital LOS, and is associated with staff and patient dissatisfaction [7,8,9,10]. Prolonged ED BT also consumes already scarce ED resources, making them unavailable for the care of new patients and potentially affecting the outcomes of non-boarded patients [1,11]. 

Despite increased recognition of the adverse effects of prolonged EDLOS, its effect on patient mortality remains unclear. Several studies have found that ED crowding and increased BT are associated with higher mortality rates [11,12,13,14,15,16]. 

Crowding can increase both EDLOS and BT, since the rate of patient intake exceeds the capacity of the triage process. Throughput is also overwhelmed, because the number of patients requiring managing is high, and a lack of hospital beds throttles patient outflow [17]. Although there is a significant relationship between crowding, boarding time, and EDLOS, the relationship with in-hospital mortality (IHM) remains unclear. 

Given the lack of evidence, additional research is needed to explore the association between EDLOS and IHM. This is important, considering recent evidence demonstrating the limited implementation and thus limited impact of hospital strategies to improve patient flow through the ED [1,2,17,18]. 

To address this knowledge gap, we performed a systematic review and meta-analysis (MA) which examined the association between EDLOS and IHM. We hypothesized that a longer EDLOS would predict greater IHM risk.

## 2. Materials and Methods

This systematic review and MA focused on studies analyzing the relationship between total EDLOS and IHM. Studies analyzing only the BT, which represents a time segment within the EDLOS (see Figure 1), and overcrowding studies that did not refer to the EDLOS were excluded. 

The review follows the 2020 Preferred Reporting Items for Systematic Reviews and Meta-Analyses (PRISMA) guidelines recommended by the Cochrane Handbook for Systematic Reviews of Interventions [19]. A PRISMA checklist is presented in Supplemental Table S1. The protocol for this review was registered in PROSPERO, CRD42016050422 (http://www.crd.york.ac.uk/PROSPERO, accessed on 29 November 2022).

### 2.1. Data Sources and Searches

We defined EDLOS as the time elapsed between the initial triage patient registration and physical departure from the ED (Figure 1). Our primary endpoint was all-cause mortality. 

A systematic search of the PubMed, Embase, Web of Science, Cochrane Controlled Register of Trials, CINAHL, PsycInfo, and Scopus databases was prepared by two medical librarians specializing in systematic reviews (L.Ö., J.C.), in close collaboration with D.L. and A.B. (emergency medicine expert physicians). All terms were searched in the fields for “Abstract” and “Article Title” (alternatively in the field for “Topic”) and MeSH/Subject Headings/Thesaurus when available. The databases were first searched from their inception to January 2020 (L.Ö.). A search update was conducted in the same databases during manuscript preparation on 14–15 January 2022 (L.Ö.), to ensure the inclusion of recently published papers. No filters or limitations were applied to retrieve the best possible results. We screened all published studies related to ED boarding and crowding to identify those reporting data on EDLOS and IHM. Studies reporting EDLOS cut-off times were included in the MA. Studies not mentioning EDLOS or IHM were excluded. We also screened the reference lists of the selected studies manually. The reviewers also manually searched the gray literature (including congress and meeting abstracts) but excluded these sources when they were not subsequently followed by full-text articles published in scientific journals. Reproducible search strings, results, and technical notes for each database are presented in Supplemental Table S2.

### 2.2. Inclusion Criteria and Study Selection

All patients over 18 years old who visited an ED were included. Exposure was defined as the time spent in the ED from the arrival to the admission to inpatient bed. This time exposure was defined as a EDLOS cut-off chosen in selected studies. The outcome was IHM whatever the cause and the delay of death in the in-hospital bed was. We considered all studies based on a prospective or retrospective design, namely cohort studies, case-control studies, as well as randomized controlled trials. 

Records identified in the literature search were uploaded to the Covidence (Veritas Health Innovation, 2021, https://www.covidence.org, accessed on 29 November 2022) systematic review software for blinded screening and automatic removal of duplicates. We extracted articles focused on the association between EDLOS and IHM in an adult ED setting. Studies analyzing the effects of boarding and ED crowding on mortality were also included when EDLOS was reported in their statistical analysis. Publications in English and other languages using translators when necessary were included. 

**Figure 2:**
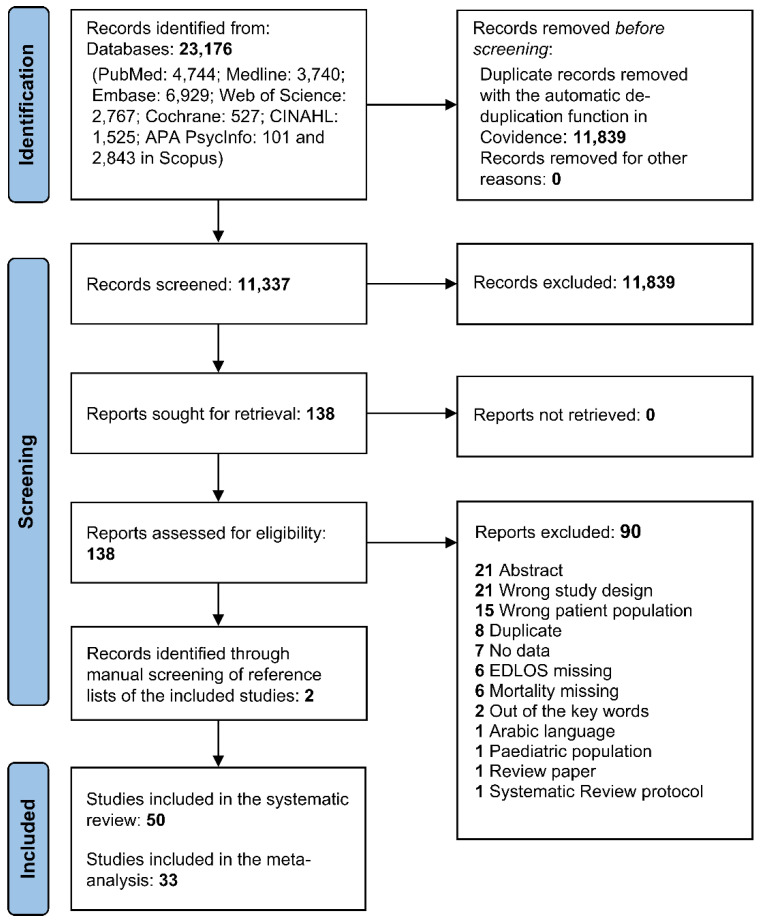
PRISMA 2020 flow diagram over the record de-duplication screening and selection process.

Two emergency medicine specialists (D.L., A.B.) independently screened the titles and abstracts yielded by the literature searches. Any selection disagreements identified by Covidence were resolved by discussion to reach consensus or were adjudicated by a third independent reviewer (Z.B.). Full reports were obtained for all titles or abstracts that met the inclusion criteria. Both reviewers independently read all full-text articles, obtaining additional information from the study authors as needed to resolve questions about eligibility. An overview of the screening and selection process is presented in the PRISMA flow diagram (Figure 2). Study data were extracted into a customized Microsoft Excel^®^ table, including the following study characteristics: design, setting, population, sample size, main objective, prognostic factors, and outcomes such as boarding, definition and values of EDLOS, crowding, type of mortality, results including precision and significance, and adjustment for confounding factors (e.g., age, comorbidities, diagnosis, triage severity code). 

### 2.3. Data Extraction and Quality Assessment 

The quality of each study was rated and recorded in a data collection form. Quality assessments were performed independently by two reviewers (A.B., Z.B.) using the Newcastle–Ottawa Quality Assessment Scale (NOS), a scale designed for non-randomized trials [20], and disagreements were resolved by discussion to reach consensus.

The NOS consists of four items on “study selection”, one item on “comparability”, and three items on “study outcome” [20]. Using this scale, reviewers can award one star for each of the four items on “selection”, one star for each of the three items on “outcome”, and a maximum of two stars for “comparability”. Ratings were calculated independently by each reviewer, and the results were averaged. Studies of the highest quality were awarded nine stars. 

The risk of bias was summarized for each study and incorporated into the overall findings and data synthesis. 

### 2.4. Data Synthesis and Analysis

The MA was performed using OpenMeta Analyst through (1) CEBM@Brown OpenMeta[Analyst] (Brown University, http://www.cebm.brown.edu/openmeta/, access on 29 November 2022), (2) Cross-platform Excel package (MetaXL, www.epigear.com, EpiGear International Pty Ltd., Castaways Beach, Noosa Heads & Sunrise Beach, Queensland, Australia), and (3) MedCalc easy-to-use statistical software package (MedCalc Software Ltd., Acacialaan, Ostend, Belgium). 

Odds ratios (ORs) were used to measure the potential association between EDLOS and IHM. For binary outcome variables, the measured effect was expressed as the log-transformed estimated OR. The weight of each study in the analysis was expressed as the inverse of the variance of the log-transformed estimated OR. The amount of between-study heterogeneity against the total variance was measured by I^2^ and presented as 0–100%.

Sensitivity analysis was performed by the leave-one-out method, in which one study at a time was removed iteratively to confirm that our findings were not dictated by any specific study. With this method, if the results are consistent, there is confidence that the overall MA results are robust. 

To illustrate the foundations, we used forest plots to summarize and visualize the effect size of each study, including 95% confidence intervals (CIs), with respect to the study’s weight. The location of the 95% CI for the OR in relation to 1, in the case of ORs, also indicated the significance of the effect size. 

We used a DerSimonian–Laird random-effects model in our study. Because the weight of each study should be approximately the same, the weighted analysis for the random-effects model was treated as an unweighted analysis. 

To examine the influence of population characteristics on overall heterogeneity, we separated the studies into two subsets for each cut-off: intensive care unit (ICU) and non-ICU population subsets. Two additional meta-analyses were conducted for each subset. 

Moreover, to improve the accuracy of our heterogeneity evaluation in the MA, we used the IVhet model in the Microsoft Excel^®^ MA package, designed particularly for use in meta-analyses with high heterogeneity (MetaXL, available at www.epigear.com, accessed on 29 November 2022) [21,22]. This method uses the quasi-likelihood estimator as an alternative to random-effects models with the problem of underestimation of the statistical error and overconfident estimates. The estimator retains a correct coverage probability and a lower observed variance than the random-effect model estimator, regardless of heterogeneity [23,24].

The symmetry of a funnel plot and Egger and Begg tests were used to qualitatively determine the presence of publication bias (MedCalc Software, version 19.6.1) [21,22].

To analyze the factors underlying heterogeneity, we performed a univariate meta-regression analysis using the following factors: age, sex, country of study, ED population, and disease severity.

## 3. Results

A total of 23,176 records were identified in the database search, with 11,337 references screened after the removal of duplicates. Two papers were added after the manual screening of the reference lists of the included papers. A search log with details and results from the search is provided in Supplemental Table S2. After screening, 50 studies were selected for inclusion in the review (Figure 2, Table 1 and Table 2) [3,9,25,26,27,28,29,30,31,32,33,34,35,36,37,38,39,40,41,42,43,44,45,46,47,48,49,50,51,52,53,54,55,56,57,58,59,60,61,62,63,64,65,66,67,68,69,70,71,72]. Thirty-three of these were included in the MA (Table 3) [25,26,27,28,29,30,31,32,33,34,35,36,38,39,40,41,42,43,44,45,46,47,48,49,50,57,58,59,60,61,63,68,71]. The remaining 17 studies [3,9,37,51,52,53,54,55,56,62,64,65,66,67,69,70,72] were excluded for one of the following reasons: (1) the specific EDLOS cut-off was not defined [3,9,37,51,52,53,54,56,62,64,65,66,67,69,72]; (2) one study reported data in severely ill mechanically ventilated patients [54]; and (3) raw data were missing in one study [70]. We subsequently acquired the original databases for four studies [34,41,42,45], which allowed us to perform analysis of nine different EDLOS cut-off values: 1.2 h [34,41,42,45,50], 1.5 h [34,40,41,42,45], 2 h [32,34,41,42,45], 3 h [34,41,42,45,47,68], 4 h [33,40,41,42,44,57,58], 5 h [32,34,38,42,46], 6 h [33,36,39,40,42,44,45,46,48,49,53,59,60,61], 8 h [25,26,27,28,29,30,31,34,41,42,45], 12 h [59], and 24 h [35,71].

### 3.1. Systematic Review 

#### 3.1.1. Characteristics of the Selected Studies

The selected studies included a total of 1,027,838 ED visits. Eleven studies were conducted in Europe (Spain [34,38], Greece [45], the U.K. [37,49], Sweden [62], Finland [47,54], The Netherlands [50], France [64], Norway [65]), 15 in North America (USA [9,30,32,33,35,36,38,40,46,55,58,60,68,72], Canada [3]), 15 in Asia (Qatar [29], Saudi Arabia [61], China [31,44,55,66], Thailand [41], India [42,72], Pakistan [48,63], Israel [57], Iran [70], South Korea [59], Turkey [69]), 7 in Australia [25,26,27,28,43,51,67], and 2 in Latin America [52,56]. The characteristics of the selected studies are listed in Table 1 and Table 2.

Two patient population types were identified across the selected studies: the non-ICU-admitted population, and the ICU-admitted population (Table 3). 

Thirty studies examined patients who were transferred from the ED to the ICU [3,25,32,34,35,36,38,39,40,42,44,45,47,48,50,51,52,53,54,55,56,58,59,60,61,63,67,69,70,71]. Twenty studies examined patients who were not admitted to the ICU, but were admitted to non-ICU wards [9,26,27,28,29,30,31,33,37,41,43,46,49,57,62,64,65,66,68,72]. 

#### 3.1.2. Non-Intensive Care Unit-Admitted Population

The non-ICU-admitted ED population was defined as a population seen in the ED and admitted to a non-critical care inpatient hospital ward (Table 3). Twenty studies analyzed the relationship between EDLOS and IHM in ED populations not admitted to the ICU [9,26,27,28,29,30,31,33,37,41,43,46,49,57,62,64,65,66,68,72]. Nine studies found an association with IHM when EDLOS exceeded a cut-off value [26,29,33,37,43,62,64,66,68], and 11 studies did not find an association [9,27,28,30,31,41,46,49,57,65,72]. Detailed information for each study is available in the Supplemental Text. 

#### 3.1.3. Intensive Care Unit-Admitted Emergency Department Population

The ICU-admitted ED population was defined as a population seen in the ED and admitted to ICU inpatient hospital ward (Table 3). Thirty studies analyzed the association between EDLOS and IHM in ED patients admitted to the ICU [3,25,32,34,35,36,38,39,40,42,44,45,47,48,50,51,52,53,54,55,56,58,59,60,61,63,67,69,70,71]. Thirteen studies found an association between EDLOS and IHM [32,38,44,45,50,55,56,59,60,61,63,70,71], while 17 did not find such an association [3,25,34,35,36,39,40,42,47,48,51,52,53,54,58,67,69]. 

Detailed information is available in the Supplemental Text.

#### 3.1.4. Quality of the Selected Studies

The methodological quality of the studies is presented in Supplemental Table S3. The evaluation was performed by two independent evaluators. The average quality score was 6.53 ± 1.23 (min.: 3; max.: 8), which can be considered intermediate.

### 3.2. Meta-analysis 

#### 3.2.1. Random-Effects Models 

**Figure 3:**
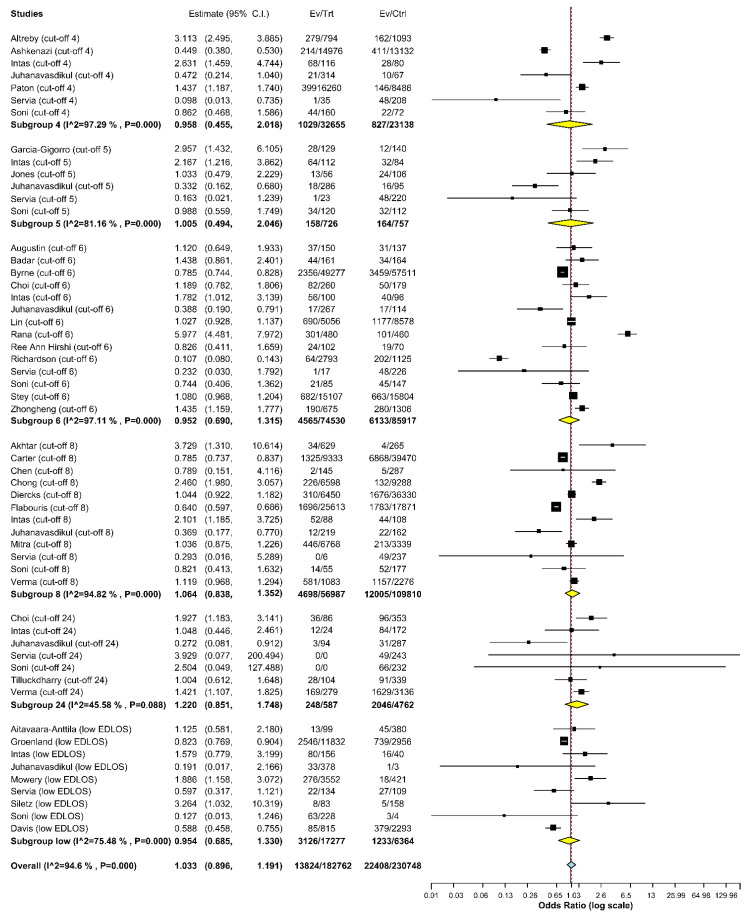
Meta-analysis including studies with the same EDLOS (1.2 h, 1.5 h, 2h, 3 h cut-offs), 4 h, 5 h, 6 h, 8 h, and 24 h cut-offs using the DerSimonian–Laird random effects model). I^2 = I^2^. EDLOS, emergency department length of stay.

The DerSimonian–Laird random-effects model [73] showed no statistically significant association between EDLOS and IHM, regardless of the cut-off value used: low EDLOS (1.2–3 h) (OR 0.954, 95% CI 0.685 to 1.330; *p* = 0.783, I^2^ = 75.481%), 4 h (OR 0.958, 95% CI 0.455 to 2.018; *p* = 0.910, I^2^ = 97.29%), 5 h (OR = 1.005, 95% CI 0.494 to 2.046; *p* = 0.989, I^2^ = 81.16%), 6 h (OR = 0.952, 95% CI 0.690 to 1.315; *p* = 0.766, I^2^ = 97.11%), 8 h (OR 1.064, 95% CI 0.838 to 1.352; *p* = 0.611, I^2^ = 94.82%), or 24 h (OR 1.220, 95% CI 0.85 to 1.748; *p* = 0.279, I^2^ = 45.58%) cut-off values (Figure 3, Supplemental Table S4). 

The ED populations included in these studies were divided into two categories: the patients admitted to the ICU (ICU-admitted population, representing the most critically ill patients) and those not admitted to the ICU (non-ICU-admitted population; those admitted to lesser-acuity in-patient wards). Our meta-analysis identified an association between EDLOS and IHM for the 24 h cut-off only in ED ICU-admitted patients, with a significant OR of 1.396 (95% CI 1.147 to 1.701, I^2^ = 0%; *p* < 0.001). Another association was found for a low EDLOS cut-off in the non-ICU-admitted ED patients’ subgroup, with a significant OR of 0.581 (95% CI 0.453 to 0.745, I^2^ = 0%; *p* < 0.001) (Supplemental Figures S1 and S2).

No significant association was found between EDLOS and IHM for any of the cut-off values when all studies, including both ICU and non-ICU populations, were tested together. After dividing the patients into the two population types to create a certain level of homogeneity in each subgroup, the effect of prolonged EDLOS on IHM could be identified. For all cut-off values, the overall effect size was close to 1, and was not statistically significant, but in the ICU subgroup, the effect size was above 1 (significant for 24 h cut-off), and in the non-ICU subgroup, the effect size was less than 1 (significant for a low EDLOS cut-off). 

#### 3.2.2. Funnel Plots

We observed a publication bias (Supplemental Figures S3–S5), as confirmed by Egger and Begg tests (Supplemental Tables S5–S7). 

#### 3.2.3. Cross-validation (Leave-one-out)

The results of the cross-validation performed by the leave-one-out method are given in Supplemental Tables S8–S10. This procedure was used in cases where insufficient data were available for partitioning between the training and test datasets. The sensitivity analysis confirmed the high heterogeneity among studies, which was not decreased by the exclusion of any single study (Supplemental Table S8).

We performed a sensitivity analysis in both ICU and non-ICU populations. The observed heterogeneity remained high in both subpopulations (Supplemental Tables S9 and S10). The exclusion of studies one by one, as suggested by Choi et al. [59], Intas et al. [45], Servia et al. [34], Soni et al. [42], Tilluckhdarry et al. [35], and Verma et al. [71], significantly reduced the heterogeneity in the ICU population for the 24 h cut-off value (Supplemental Table S9). Sensitivity analysis for the non-ICU population was possible only for a cut-off of 4 h, with the exclusion of the Paton et al. study (Supplemental Table S10) [44]. 

To summarize, for most cut-off values except for EDLOS <3 h and EDLOS >24 h, in the studies overall and in the ICU and non-ICU subgroups separately, no single study had a significant effect on the test results. 

#### 3.2.4. Inverse Variance Heterogeneity Model 

Because of the high level of heterogeneity between studies, we decided to conduct a meta-analysis using the inverse variance heterogeneity (IVhet) model [23,24]. We did not find a significant difference in IHM between patients staying in the ED for any of the investigated cut-off periods (Supplemental Table S11). The use of the IVhet model allows reducing the underestimation of the statistical error and overconfident estimates. In all cases, even if the 95% CI for the effect size of the random-effects model revealed a significant result, the IVhet model provided a broader 95% CI for the same effect size, so that eventually, none of the effect sizes were statistically significant. This finding supported the main conclusion that there is no significant association between EDLOS and IHM. 

#### 3.2.5. Subgroup Meta-Analyses and Univariate Meta-Regression Analysis 

We performed different meta-analyses to isolate subpopulations to explain the observed high heterogeneity. First, we excluded step-by-step studies because we observed that this exclusion decreased heterogeneity. The random-effects model confirmed the absence of an association (Supplemental Table S12). Next, we performed meta-analyses of studies that included the general ED population (Supplemental Table S12), specific disease populations, and patients with different severities of illness (ICU and non-ICU populations) (Supplemental Table S12). We found that the disease population and severity of illness were involved in the heterogeneity (Supplemental Table S12).

To explain the source of heterogeneity, a univariate meta-regression analysis was performed for each cut-off value separately. As expected, some of the factors had a significant effect on heterogeneity. For example, in meta-regression analysis for the 6 h cut-off, all the factors were significant at a 5% significance level.

## 4. Discussion

EDLOS and BT are used by hospital administrators as measures of the quality of care delivered in the ED. A prolonged EDLOS is a source of dissatisfaction for patients and family; however, this indicator in isolation is not sufficient to comprehensively evaluate the quality of care. Combining ED time and the occurrence of negative outcomes, such as adverse events and IHM, is comparatively more relevant, and could help to improve quality of care. We previously found that there was a trend that BT increases IHM [74]. This new systematic review and MA did not find a significant relationship between EDLOS and IHM for any of the studied cut-off time points. However, our research did uncover a new and relevant result for EDLOS >24 h in ED ICU-admitted patients and EDLOS <3 h in non-ICU-admitted ED patients. For these cut-offs and types of ED populations, we did not find heterogeneity (I^2^ = 0). The absence of a statistically significant difference in IHM for the other cut-offs is likely multifactorial, including the heterogeneity among the studies and various other factors, including population characteristics (e.g., age, sex, triage severity score, type of disease, mode of arrival at the ED, ED daytime, time shift, etc.), variation in hospital organization, adherence to clinical guidelines, type of admission source, and other factors. We used IVhet, designed particularly for use in meta-analyses with high heterogeneity, to provide better validation for the same estimated effect size [23,24]. Regular random-effects models, such as inverse variance or DerSimonian–Laird [73,75,76,77], emphasize the need for larger studies and indicate an underestimation of the statistical error. However, the IVhet model provides the correct coverage of the estimated effect size. The CI of the effect size obtained with this model was wider than that in other random-effects models. All 95% CIs using the IVhet model included 1; thus, we can conclude that there was no significant association between EDLOS and IHM for cut-off values of 4–8 h, which represent the target times in some countries [4,5,6]. Cross-validation analysis did not reduce the heterogeneity (Supplemental Table S11). However, meta-regression analysis showed that factors, such as type of population, type of disease, and severity of illness, could explain the heterogeneity for EDLOS <3 h, 4 h, 5 h, and 6 h cut-off values (Supplemental Table S12). Most categorical variables (e.g., population type, severity score, and country) were found to be significant in at least some of the meta-regression models at different cut-offs. However, there was no consistent impact of one variable on all cut-offs.

In exploring this lack of association between EDLOS and IHM for some cut-offs, we recognize that processing time and patient care time are complex variables, combining many different factors that influence the EDLOS, quality of care, and patient safety in the ED [78,79]. Given the frenzied nature of the ED environment, crowding may prevent providers from giving critically ill patients the close and constant attention they need [80,81,82,83,84,85,86,87,88,89,90,91,92]. This could be expected to lead to worse outcomes for patients, including increased IHM, but the evidence that we found in this systematic review was mixed. While some studies suggested that EDLOS is an independent predictor of ICU mortality [3,25,34,35,36,39,40,42,47,48,51,52,53,54,58,67,71], others reported no adverse association [32,38,44,45,50,55,56,59,60,61,63,69,70]. MA of the studies reporting IHM in patients admitted to the ICU showed an association with EDLOS over 24 h, with absence of heterogeneity. In most EDs, it is only acceptable to keep critical patients in the ED when there are no ICU beds. Many EDs are not designed to manage those patients optimally, due to a lack of trained emergency specialists in some countries, a lack of nurse resources, or the absence of a specific intensive care area where critical patients can be safely observed by a specific team. In ED patients who were admitted to non-ICU wards, some studies showed an association between EDLOS and IHM [26,29,33,37,43,62,64,66,68], while other studies did not [9,27,28,30,31,41,46,49,57,65,72]. Surprisingly, with cut-off values analyzed through different random-effects meta-analyses, we found a significant association between EDLOS < 3 h and IHM. Our data do not provide an explanation of this finding, and prospective studies analyzing all factors that contribute to the EDLOS are needed. It is typically rare to admit ED patients within 3 h, which is often below the threshold for obtaining all laboratory and imaging results, and for some patients’ specialist consultations. Sicker patients and those with clear-cut diagnoses who receive certain specific treatments may account for early departures, which could explain this result.

Our recommendations for policy makers are that long stays in the ED must be discouraged, unless there is a specific track for these patients including a specific ED area with a dedicated team. Another lesson from our study is that ED teams must be careful when they admit patients after a short stay in the ED and should be sure that there is continued close monitoring to avoid the risk of clinical deterioration. This may be particularly true in older patients where clinical presentations are often atypical. Some critically ill patients will be treated in the ED [58]. In such cases, the outcome and EDLOS will be dependent on the rapidity to stabilize the patients and the decision to admit them to hospital [57].

Another recommendation is to fast-track the care of specific events, such as myocardial infarction or stroke, that will be directly addressed to the angiography laboratory and acute neurovascular unit, resulting in a markedly reduced EDLOS for these patients. The association between EDLOS at different cut-off time points and IHM at different hospital time points (24 h, 48 h, 72 h, 7 days, 28 days), including the occurrence of adverse events after the ED care, could be worth investigating.

In contrast to high priority/sicker patients, mortality in patients with lower triage could be positively associated with EDLOS. One explanation could be the effect of under-triaging, where patients with medical urgency remain undetected by the ED triaging system. Patients with non-specific symptoms and low clinical urgency often have increased hospitalization, increased EDLOS, increased mortality, and more often are frail and of advanced age [9,93].

### 4.1. Study Strengths

The strengths of this MA include an extensive comprehensive search strategy, strong eligibility criteria that enhance generalizability, rigorous use of the NOS approach for rating the quality of evidence, a robust step-by-step MA, and a large number of included studies. This is the first MA exploring the association between EDLOS and IHM. 

### 4.2. Study Limitations

Our study had some limitations and potential presence of publication bias. Some studies included a univariate analysis, while others used multivariate analysis, making it difficult to compare the effect sizes. Therefore, we chose to use an overall univariate analysis using the crude data reported by the studies. We used various meta-analyses and used the IVhet method to confirm the absence of an association for the classical cut-offs observed in the EDs. Moreover, a dose–response meta-analysis model was inapplicable for the relationship of EDLOS and IHM, given the lack of sufficient EDLOS-specific comparisons within each included study (i.e., only two-time ranges in each study) [94]; a dose–response model would be useful to determine the golden time range of EDLOS for patients needing emergency healthcare, and meanwhile explain the heterogeneity of the results. In addition, our study explored the association between EDLOS and IHM, but did not address the causes of prolonged EDLOS. With 33 studies from 50 countries worldwide included in the systematic review, we believe the results are generalizable to larger, urban, academic EDs, which represent the vast majority of EDs contributing to this MA. Representation of smaller, rural, non-academic EDs is limited, and therefore, generalizability to these EDs is unclear. More studies are needed to evaluate the correlation between EDLOS and IHM in different countries and hospital types, with variable equipment and human resources to confirm the results for EDLOS <3 h and EDLOS >24 h. In addition, a better understanding of the role played by potential confounding factors can help to reduce heterogeneity for the other cut-offs.

## 5. Conclusions

This MA was designed to analyze the association between EDLOS and IHM; we did not find evidence supporting this hypothesis when all ED patients were included for each cut-off. However, we did find a new and relevant result confirming an association with EDLOS and IHM for patients exceeding 24 h in ED ICU-admitted patients and for low EDLOS below 3 h in non-ICU-admitted ED patients. Other factors involved in the negative outcomes after ED care should be carefully explored to determine the role of EDLOS in the occurrence of IHM.

## Figures and Tables

**Table 1:** Characteristics of the selected studies for the systematic review.

N	Author	Year	Journal	Country and ED Setting	EDLOS Cut-Off
1	Carter AW [25]	2010	Emerg Med Australas	45 Australian hospitals	Qualitative (8 h)
2	Mitra B [26]	2012	Intern Med J	3 Australian hospitals	Qualitative (8 h)
3	Chong CP [27]	2013	Australas J Ageing	1 Australian hospital	Qualitative (8 h)
4	Flabouris A [28]	2013	Emerg Med Australas	1 Australian hospital	Qualitative (8 h) and continuous
5	Akhtar N [29]	2016	J Stroke Cerebrovasc Dis	1 Qatari hospital	Qualitative (8 h)
6	Diercks DB [30]	2007	Ann Emerg Med	550 U.S. hospitals	Qualitative (<4 h, 4–8 h, >8 h)
7	Chen HC [31]	2016	Intern Emerg Med	1 Chinese hospital	Qualitative (8 h)
8	Jones EM [32]	2015	J Crit Care Med	1 U.S. hospital	Qualitative (5 h)
9	Mowery NT [33]	2011	J Trauma	The U.S. healthcare system	Qualitative (2 h) and continuous
10	Serviá L [34]	2012	J Crit Care	1 Spanish hospital	Qualitative (2 h)
11	Tilluckdharry L [35]	2005	Am J Emerg Med	1 U.S. hospital	Qualitative (24 h)
12	Hirshi RA [36]	2018	Adv Emerg Nurs J	1 U.S. hospital	Quantitative, continuous No specific EDLOS cut-off
13	Plunkett PK [37]	2010	Eur J Em Med	1 U.K. hospital	Qualitative with multiple cut-offs: 2.6 h, 3.9 h, 5.8 h, and 8.7 h No specific EDLOS cut-off
14	García-Gigorro R [38]	2016	Med Intensiva	1 Spanish hospital	Qualitative (6 h)
15	Agustin M [39]	2017	Crit Care Res Pract	1 U.S. hospital	Qualitative (6 h)
16	Siletz A [40]	2017	J Surg Res	1 U.S. hospital	1.5 h
17	Junhasavasdikul D [41]	2013	Em Med J	1 Thailand hospital	No specific EDLOS cut-off
18	Soni KD [42]	2018	J Emerg Trauma Shock	1 Indian hospital	No specific EDLOS cut-off
19	Paton A [43]	2018	Emerg Med Australas	3 Australian hospitals	4 h
20	Zhang Z [44]	2019	Em Med J	1 Chinese hospital	Qualitative (<6 h, 6–12 h, 12–24 h, >24 h)
21	Intas G [45]	2012	Adv Emerg Nurs J	1 Greek hospital	Qualitative (6 h)
22	Richardson JD [46]	2009	J Am Coll Surg	1 U.S. hospital	6 h
23	Aitavaara-Anttila M [47]	2019	Acta Anaesthesiol Scand	1 Finnish hospital	3 h
24	Khan BA [48]	2016	J Pak Med Assoc	1 Pakistani hospital	6 h
25	Byrne D [49]	2018	Acute Med	1 U.K. hospital	6 h
26	Groenland CN [50]	2019	Crit Care Med	6 university hospitals in The Netherlands	1.2 h
27	Haji K [51]	2010	Crit Care Shock	1 Australian hospital	Continuous No specific EDLOS cut-off
28	Santos FR [52]	2020	Rev Bras Ter Intensiva	1 Brazilian hospital	Continuous No specific EDLOS cut-off
29	Mejaddam AY [53]	2013	J Emerg Med	1 U.S. hospital	Continuous No specific EDLOS cut-off
30	Saukonnen KA [54]	2006	J Intern Med	1 Finnish hospital	Continuous No specific EDLOS cut-off
31	Hung SC [55]	2014	Crit Care	1 Chinese hospital	4 h
32	Cardoso LT [56]	2011	Crit Care	1 Brazilian hospital	Continuous No specific EDLOS cut-off
33	Ashkenazi [57]	2021	Am J Em Med	28 Israeli hospitals	4 h
34	Stey [58]	2021	J Int Care Med	4 U.S. hospitals	6 h
35	Choi [59]	2021	Emerg Med Intern	5 Korean hospitals	6 h 12 h 24 h
36	Lin [60]	2021	QJM: An International Journal of Medicine	2 U.S. hospitals	6 h
37	Altreby [61]	2021	Rev Bras Ter Intensiva.	1 Saudi Arabian hospital	4 h
38	Wessman [62]	2021	Internal and Emergency Medicine	1 Swedish hospital	No cut-off
39	Rana [63]	2021	Pakistan Journal of Medical and Health Sciences	1 Pakistani hospital	6 h
40	Thibon [64]	2019	Ann. Fr. Med. Urgence	1 French hospital	No cut-off
41	Asheim [65]	2019	European Journal of Emergency Medicine	1 Norwegian hospital	No cut-off
42	Cheng [66]	2022	World J Emerg Med	1 Chinese hospital	No specific cut-off
43	Crilly [67]	2019	Emergency Medicine Australasia	1 Australian hospital	No specific cut-off
44	Davis [68]	2021	Journal of Neuroscience Nursing	U.S. hospitals	3 h
45	Elay [69]	2020	Eurasian J Emerg Med.	1 Turkish hospital	No specific cut-off
46	Rose [3]	2016	Annals ATS	Canadian hospitals	6 h
47	Sabaz [70]	2020	Iran Red Crescent Med J.	1 Iranian hospital	No specific cut-off
48	Verma [71]	2021	Indian Journal of Critical Care Medicine	1 Indian hospital	8 h and 24 h
49	Derose [9]	2014	Med Care	14 U.S. hospitals	No specific cut-off
50	Jain [72]	2013	Western J Emerg Med	1 U.S. hospital	No specific cut off

EDLOS, Emergency Department Length of Stay.

**Table 2:** Factors analyzed and association between EDLOS and in-hospital mortality in the selected studies for the systematic review.

N	Author	Type of ED Population	Study Group (n)	Statistics	Factors Analyzed	Association with IHM
1	Carter [25]	ICU	48,803	Logistic regression Adjusted	Age, comorbidity, source of admission, year of admission, number of admissions to ICU from ED per day, type of hospital, diagnosis, MV, acute renal failure	No
2	Mitra [26]	Non-ICU	10,107	Logistic regression Adjusted	Age, sex, triage category, time to disposition plan	Yes
3	Chong [27]	Non-ICU	15,886	Logistic regression adjusted	Age, sex, comorbidities	No
4	Flabouris [28]	Non-ICU	43,484	Backwards stepwise regression	Age, EDLOS, sex, admission source, admitting unit, ED arrival time and day of week, ED departure time, Australasian triage scale	No
5	Akhtar [29]	Non-ICU	894	Logistic regression Adjusted	Sex, Health Technology Assessment, atrial fibrillation, smoking, thrombolysis complications, urinary tract infection, bedsores, Foley’s catheter	Yes
6	Diercks [30]	Non-ICU	42,780	Logistic regression Adjusted	Age, sex, Body Mass Index, race, insurance, cardiac risk factor, past cardiac history	No
7	Chen [31]	Non-ICU	432	No logistic regression	Age, sex, comorbidities, renal function, cardiac biomarkers, systolic and diastolic blood pressure, heart rate, time of primary PCI, door to balloon, advanced heart failure, Killip score, TIMI risk score, respiratory failure, anterior wall STEMI, any post myocardial complications, left ventricular ejection	No
8	Jones [32]	ICU	162	Logistic regression Adjusted	Age, sex, Baseline Intracerebral Hemorrhage Score (age, GCS, intracerebral hemorrhage volume and location, intraventricular hemorrhage)	Yes
9	Mowery [33]	Non-ICU	3973	Logistic regression Adjusted	Age, sex, ISS, revised trauma score	Yes
10	Serviá [34]	ICU	243	Logistic regression Adjusted	Age, sex, mechanical ventilation, head injury with AIS ≥ 3, TRISS ≥ 20	No
11	Tilluckdharry [35]	ICU	443	No logistic regression Not adjusted	Age, sex, disease, APACHE II score	No
12	Hirshi [36]	ICU	294	Logistic regression	Arrival by emergency medical services, septic shock, liver disease, baseline lactate, Sequential Organ Failure Assessment, time to antibiotics and fluids, number of vasopressors	No
13	Plunkett [37]	Non-ICU	23,114	Logistic regression Adjusted	Sex, Acute Illness Severity Score, triage score category, major disease by category, Charlson’s comorbidity index, ICU admission, blood transfusion, troponin elevation, door-to-team, and team-to-ward time	Yes
14	García-Gigorro [38]	ICU	269	Logistic regression	Age, sex, comorbidities, diagnostic category, APACH score	Yes
15	Agustin [39]	ICU	287	Logistic regression	SOFA, MAP, and lactate	No
16	Siletz [40]	ICU	241	Logistic regression	Age, ISS, number of comorbidities	No
17	Junhasavasdikul [41]	Non-ICU	381	Logistic regression	Age, sex, primary diagnosis, lead-time, severity triage score, MEWS	No
18	Soni [42]	ICU	232	Logistic regression	Age, sex, SpO_2_, GCS, ICU stay days, heart rate, referring, status, ISS	No
19	Paton [43]	Non-ICU	24,746	Logistic regression	Sex, age, triage, category, ambulance transport, residing at home	Yes
20	Zhang [44]	ICU	1997	Logistic regression	PaO2/FiO2, serum creatinine, age, SOFA, Body Mass Index, lactate, comorbidities and infection site	Yes
21	Intas [45]	ICU	200	Logistic regression	Age, sex, diagnostic category (e.g., medical, surgical), APACH score, SAOS II, GCS at the time of intubation, admission time	Yes
22	Richardson [46]	Non-ICU	3918	Logistic regression	Age, mechanism of injury, race, sex, GCS, computed tomography findings of the head, abdomen, and chest, ISS,	No
23	Aitavaara-Anttila [47]	ICU	479	Logistic regression	NEWS on ED admission, SOFA score, and APACH score on ICU admission, GCS, urine output, blood pressure, oxygen saturation, respiratory rate, pulse rate, body temperature, use of oxygen or form of mechanical ventilation, use of vasoactive medication, chronic illnesses, and medications	No
24	Khan [48]	ICU	325	Logistic regression	Age, sex, time of presentation, ED triage category, vital signs, presenting complaints, comorbid conditions, laboratory values, radiological studies, procedures, severity of illness scores	No
25	Byrne [49]	Non-ICU	106,788	Logistic regression	Age, sex, severity triage score, illness severity score, comorbidities, sepsis, disabilities	No
26	Groenland [50]	ICU	14,788	Logistic regression	Disease severity, APACH IV score, comorbidities, age, admission diagnosis, reason for ICU admission (medical, urgent, or elective)	Yes
27	Haji [51]	ICU	117	Logistic regression	Age, sex, APACH II score, physiological and biochemical data: heart rate, respiratory rate, temperature, systolic blood pressure, GCS, pH, partial pressure of carbon dioxide, serum bicarbonate, white cell count, time to resuscitation and time to antibiotics	No
28	Santos [52]	ICU	6176	Logistic regression	Age, sex, admission due to neurological disease, cancer, infectious disease, hypertension, level of dependency, chronic dialytic kidney disease, GCS on admission, SOFA score, dependency level, use of vasopressors, mechanical ventilation, need for renal replacement therapy	No
29	Mejaddam [53]	ICU	224	Logistic regression	Age, sex, time and date of presentation, mechanism of injury, current use of antithrombotic medications, timing of intubation, initial results of radiological imaging, head computed tomography results, GCS, initial pupil reactivity, administration of blood products, initial laboratory values, initial vital signs, intracranial pressure monitor placement, use of antiepileptic agents, hyperosmolar agents, and vasopressors, prolonged hypotension	No
30	Saukonnen [54]	ICU	1675	No logistic regression	Age, sex, place of admission, NYHA class, diagnoses according to the APACH II score, and ICD (Tenth Revision), SAPS II, SOFA, TISS score	No
31	Hung [55]	ICU	1242	Logistic regression	Age, sex, vital signs, triage results, chief complaints, laboratory findings, baseline comorbidities, hospital discharge condition, length of ventilator use, APACH II score, diagnostic categories	Yes
32	Cardoso [56]	ICU	401	Cox regression	Age, sex, APACH II score, comorbidities, need for mechanical ventilation and tracheal intubation, vasoactive drug use, TISS score, SOFA, hospital ward admission	Yes
33	Ashkenazi [57]	Non-ICU	28,108	Logistic regression	Age, sex, type of specialty ward admission	No
34	Stey [58]	ICU	30,915	Logistic regression	Age, sex, race, systolic blood pressure, oxygen saturation, GCS, comorbidities (diabetes, cerebrovascular accident, dementia, dependent functional status, cirrhosis, varices), injuries (intracerebral hemorrhage, contusion, lung injury) insurance, transfer status, emergency transport vehicle, admission year, teaching hospital status, ACS trauma center designation, number of orthopedic and trauma surgeons and number of trauma ICU beds.	No
35	Choi [59]	ICU	439	Logistic regression	Age, sex, comorbidities (hypertension, diabetes, chronic renal disease, cardiovascular disease, and malignancy status), initial vital signs (systolic blood pressure, diastolic blood pressure, pulse rate, respiratory rate, and body temperature), KTAS level in the ED, SOFA score.	Yes
36	Lin [60]	ICU	13,634	Logistic regression	Age, sex and SAPS II, covariates were adjusted for age, sex, ethnicity, marital status, length of ICU stay, length of hospital stay, ICU types, SAPS II, and diagnostic category.	Yes
37	Altreby [61]	ICU	1887	Logistic regression	Age, sex, diagnosis, and general diagnostic category (medical, surgical, and trauma), mechanical ventilation status, need for vasopressors, need for CRRT, insertion of a central venous line, measures of severity such as APACHE IV, SOFA score, MEWS, and sepsis status.	Yes
38	Wessman [62]	Non-ICU	641,314	Logistic regression	Age, sex, any of the ten most common chief complaints pre-defined (abdominal pain, chest pain, shortness of breath, painful or swollen extremity, malaise, dysrhythmia, allergic reaction, syncope, intoxication, fever and undefined), triage priority at arrival, if the patient was given prehospital care given by ambulance or not, if the patients were admitted to in-hospital care or not if the patient presented to the ED in the weekend or not. The chief complaints can be seen as a crude proxy for comorbidity and should eliminate some confounding associated with complaint.	Yes
39	Rana [63]	ICU	460	No logistic regression	Time of admission, primary diagnoses, co-morbidities, time spent in ED from presentation to reaching ICU, APACHE IV.	Yes
40	Thibon [64]	Non-ICU	15,496	No logistic regression	Age, sex, severity triage score, biology, imaging	Yes
41	Asheim [65]	Non-ICU	165,183	Logistic regression	Age, sex, cardiovascular disease, infection, medical specialty, arrival with ambulance	No
42	Cheng [66]	Non-ICU	4972	Logistic regression	Age, sex, time of arrival, arrival with ambulance, ward disposition, number of ED patients, disease categories, health insurance	Yes
43	Crilly [67]	ICU	423	Logistic regression	Age, sex, severity triage score, daytime, time to the ED, mode of arrival, diagnosis, APACHE score	No
44	Davis [68]	Non-ICU	3108	Logistic regression	Age, sex, NIH-SSS score, comorbidities	Yes
45	Elay [69]	ICU	206	No logistic regression	Age, sex, disease severity scores, comorbidities, antibiotic administration, blood culture results, length of hospital stay, and 30-day mortality. SOFA and APACHE II	No
46	Rose [3]	ICU	314,836	Logistic regression	Age, sex, severity triage score, diagnosis, comorbidities, ventilation, ED annual census, ED shift time, institution, specialized center, hospital occupancy, WE admission	No
47	Sabaz [70]	ICU	1297	Logistic regression	Age, sex, length of hospital stay, length of ICU stay, ICU admission diagnosis, APACHE II score and comorbidities, APACHE II score, APACHE IV score, SAPS 3 score, SOFA score, TISS score, GSC score on the first day of ICU, results of blood samples taken on the first day of ICU, need for MV and tracheal intubation, vasoactive agents use, invasive procedures used, treatments	Yes
48	Verma [71]	ICU	3429	No logistic regression	Age, sex, diagnosis, severity triage score	Yes
49	Derose [9]	Non-ICU	136,740	Logistic regression	Age, sex, severity triage score, ambulance arrival, race, ED shift time, blood pressure, heart rate, ED system crowding	No
50	Jain [72]	Non-ICU	190	Logistic regression	Age, sex, NIH-SSS, disposition, hospital length of stay, comorbidity, thrombolysis	No

AIS, Abbreviated Injury Score; ACS, American College of Surgeons; APACH, Acute Physiology and Chronic Health Evaluation; CRRT, continuous renal replacement therapy; ED, emergency department; EDLOS, ED length of stay; GCS, Glasgow Coma Scale; ICU, intensive care unit; Injury Severity Score, ISS; In-Hospital Mortality, IHM; International Classification of Diseases, ICD; KTAS, Korean Triage and Acuity Scale; MAP, mean arterial blood pressure; MEWS, Modified Early Warning Score; MV, mechanical ventilation; NEWS, National Early Warning Score; NIH-SS, NIH Stroke Scale; RTS, Revised Trauma Score; SAPS, Simplified Acute Physiology Score II; TRISS, therapeutic intervention scoring system; SOFA, Sequential Organ Failure Assessment; TISSS, Therapeutic Intervention Scoring System Score.

**Table 3:** Distribution of the studies that showed an association between EDLOS and IHM for each population and quality score of studies.

	Author	Study Group (n)	Association with IHM	Quality Score of Studies	Confounding Factors	MA
ICU	Carter [25]	48,803	No	8	Not analyzed	Yes
	Jones [32]	162	Yes	6	Not analyzed	Yes
	Serviá [34]	243	No	7	Not analyzed Factors associated with mortality: age greater than 60 years, MV, head injuries with abbreviated injury scale scores of 4 or higher, and shock	Yes
	Tilluckdharry [35]	443	No	8	Not analyzed	Yes
	Hirshi [36]	294	No	8	Not analyzed Factors associated with mortality: liver disease	Yes
	García-Gigorro [38]	269	Yes	5.5	Not analyzed	Yes
	Agustin [39]	287	No	5	SOFA, mean arterial blood pressure, and lactate	Yes
	Siletz [40]	241	No	6	Age, ISS, number of comorbidities	Yes
	Soni [42]	232	No	3.3	Age, SpO2, GCS, referring status, ICU stay	Yes
	Zhang [44]	1997	Yes	6	PaO2/FiO2, serum creatinine, age, SOFA, Body Mass Index, lactate, comorbidities, and infection site	Yes
	Intas [45]	200	Yes	6	Reason for admission (surgical vs. medical), direct versus indirect ICU admission, time of admission, fever	Yes
	Aitavaara-Anttila M [47]	479	No	8	Not analyzed	Yes
	Khan [48]	325	No	6	Age, discharge diagnostic (renal, sepsis, malignancy, respiratory), CT scan result, triage category	Yes
	Groenland [50]	14,788	Yes	8	APACH IV, comorbidities, age, admission diagnosis, reason for ICU admission	Yes
	Haji [51]	117	No	6	Not analyzed	No
	Santos [52]	6176	No	6	Age, sex, neurological disease, infection/sepsis, cancer, arterial hypertension, need for assistance, chronic renal dialysis, GCS at admission	No
	Lin [60]	13,634	Yes	6	ICU types, length of hospital stay, length of ICU stay, SAP score II, diagnostic category	Yes
	Mejaddam [53]	224	No	6	Not analyzed No clear mortality data	No
	Saukonnen [54]	1675	No	6	No analyzed	No
	Hung [55]	1242	Yes	6	Higher APACHE II score, triage level as non-urgent, sex, diagnostic category	No
	Cardoso [56]	401	Yes	6	Sex, age, comorbidity, APS, SOFA, TISS, general hospital ward, sepsis diagnosis	No
	Stey [58]	30,915	No	7	ACS verification hospital level, adult beds, triage score, hospital teaching status, trauma ICU beds, number of neurosurgeons, number of orthopedic surgeons, number of trauma surgeons	Yes
	Choi [59]	439	Yes	6.5	Malignancy, systolic blood pressure, platelets, albumin, SOFA, septic shock, vasopressor at ED, ventilator at ED	Yes
	Altreby [61]	1887	Yes	6.5	Age, sex, mechanical ventilation, CRRT, vasopressors, central venous line, diagnosis, APACHE IV, SOFA, MEWS, sepsis, time to admission, ICU length of stay	Yes
	Rana [63]	460	Yes	5.5	Not analyzed	Yes
	Crilly [67]	423	No	5.5	Not analyzed	No
	Elay [69]	206	No	5	Not analyzed	No
	Rose [3]	314,836	Yes	7	Age, sex, comorbidity, trauma admission, ventilation, ED annual census, ED shift time, institution, specialized center, hospital occupancy, weekend admission, ICU census	No
	Sabaz [70]	1297	No	6.5	MV, lactate, APACH 2, SAP 3, APACH 4, SOFA, inotrope agent, septic shock warning, white blood count	No
	Verma [71]	3429	Yes	6	Not analyzed	Yes
Non-ICU	Mitra [26]	10,107	Yes	6	Age, sex, triage category, hospital type	Yes
	Chong [27]	15,886	No	8	Age, injuries, sepsis, stroke, pneumonia, renal diseases, COPD, liver diseases	Yes
	Flabouris [28]	43,484	No	7	Age, triage category, sex, admission source	Yes
	Akhtar [29]	894	Yes	7	Age, prior stroke, coronary artery disease, history of smoking, dysphagia present at admission	Yes
	Diercks DB [30]	42,780	No	7	Not analyzed	Yes
	Chen [31]	432	No	7	Not analyzed	Yes
	Mowery [33]	3973	Yes	8	RTS, age, ISS,	Yes
	Plunkett [37]	23,114	Yes	6	Sex, major disease by category, Charlson’s comorbidity index, ICU admission, blood transfusion, troponin elevation	No
	Junhasavasdikul [41]	381	No	4	MEWS, sepsis, Eastern Cooperative Oncology Group	Yes
	Paton [43]	24,746	Yes	6	Age, sex, triage category, ambulance transport, residing at home	Yes
	Richardson [46]	3918	No	6	Age, ISS, GCS, positive CT	Yes
	Byrne [49]	106,788	No	8	Not analyzed	Yes
	Ashkenazi [57]	28,108	No	8	Age, sex	Yes
	Wessman [62]	641,314	Yes	7.5	Age	No
	Thibon [64]	15,496	Yes	6.5	Not analyzed	No
	Asheim [65]	165,183	No	7	Age, sex, cardiovascular disease, infection, ambulance, medical specialty	No
	Cheng [66]	4972	Yes	6.5	Not analyzed	No
	Davis [68]	3108	Yes	8	Age, comorbidity	Yes
	Derose [9]	136,740	No	5	Sex, race/ethnicity, and pre-existing comorbidities, ambulance arrival, triage, blood pressure and pulse, triage score, diagnosis, day shift, weekend, month	No
	Jain [72]	190	No	8	SS, thrombolysis, hospital length of stay	No

The average study quality score is 6.48 (max.: 8, min.: 3.5) for the ICU-admitted population and 6.45 (max.: 8, min.: 4) for the non-ICU population. The Newcastle–Ottawa Quality Assessment Scale was used to evaluate the quality of the studies [20]. ACS, American College of Surgeons; APACH, Acute Physiology and Chronic Health Evaluation; CRRT, continuous renal replacement therapy; ED, emergency department; EDLOS, emergency department length of stay; GCS, Glasgow Coma Scale; ICU, intensive care unit; IHM, in-hospital mortality; ISS, Injury Severity Score; MA, meta-analysis; MEWS, Modified Early Warning Score; MV, mechanical ventilation; SAPS, Simplified Acute Physiology Score II; SOFA, Sequential Organ Failure Assessment; SS, Stroke Severity; TISSS, Therapeutic Intervention Scoring System Score.

## Data Availability

Template data collection forms, data extracted from included studies, data used for all analyses, analytic code, and any other materials used in the review are available from the corresponding author upon request. We also utilized de-identified data pertaining to EDLOS cut-offs and mortality that did not require ethical committee approval.

## Supplementary Materials

The following supporting information can be downloaded at: https://www.mdpi.com/article/10.3390/jcm12010032/s1, Supplemental Text. Detailed information for each selected study; Figure S1. Meta-analysis of studies including the ICU-admitted ED population with the same cut-off using the random-effects model (DerSimonian–Laird), I^2 = I^2^; Figure S2. Meta-analysis of studies including the non-ICU-admitted ED population with the same cut-off using the random-effects model (DerSimonian–Laird), I^2 = I^2^; Figure S3. Funnel plots for all studies for each cut-off. (A) Low EDLOS (1.2 h, 1.5 h, 2 h, 3 h), (B) 4h cutoff, (C) 5h cut-off, (D) 6h cut-off, (E) 8h cut-off, (F) 24h cut-off; Figure S4. Funnel plots for ICU-admitted population. (A) Low EDLOS (1.2 h, 1.5 h, 2 h, 3 h), (B) 4 h cut-off, (C) 5 h cut-off, (D) 6 h cut-off, (E) 8 h cut-off, (F) 24 h cut-off; Figure S5. Funnel plots for non-ICU-admitted population. (A) 4 h cut-off, (B) 4 h cut-off, (C) 6 h cut-off, (D) 8 h cut-off. The remaining groups include 1–2 studies only; Table S1 [95]. PRISMA 2009 checklist for the systematic review and meta-analysis; Table S2. Search specifications for PubMed, Medline, Embase, Web of Science, Cochrane Library, CINAHL, APA PsycInfo, and Scopus; Table S3. Quality assessment of the studies. The Newcastle–Ottawa Quality Assessment Scale consists of 4 items on study selection, 1 item on comparability, and 3 items on study outcomes (see Reference 20 in main manuscript). According to this scale, studies can be awarded one star for each of the 4 items on selection and for each of the 3 items on outcomes, and a maximum of 2 stars for comparability. The highest quality studies are awarded up to nine stars. The evaluation was performed by two independent evaluators (E1 and E2). The average is 6.53 ± 1.23 (min.: 3; max.: 8); Table S4. Summary of the random-effects model results (DerSimonian–Laird); Table S5. Publication bias tests for all cut-offs; Table S6. Publication bias tests for all cut-offs in the ICU population; Table S7. Publication bias tests for all cut-offs in the non-ICU population; Table S8. Sensitivity analysis (leave-one-out) results of the overall data. Random-effects model (DerSimonian–Laird); Table S9. Sensitivity analysis (leave-one-out) results of the ICU population. Random-effects model (DerSimonian–Laird); Table S10. Sensitivity analysis (leave-one-out) results of the non-ICU population. Random-effects model (DerSimonian–Laird); Table S11. Heterogeneity analysis: comparison between inverse variance (IV) and inverse variance heterogeneity (IVhet) methods. Estimated effect size and CI (95%); Table S12: Meta-regression analysis. Discrete (categorical) factors are population type, country, and number of patients included in the studies. The continuous factors are age and sex. Discrete (categorical) factors = population type, severity (ICU/not ICU), country, disease. Continuous factors = age and % male.

## Abbreviations

AIS	Abbreviated Injury Score
ACS	American College of Surgeons
APACH	Acute Physiology and Chronic Health Evaluation
BT	Boarding time
CRRT	Continuous renal replacement therapy
CI	Confidence interval
ED	Emergency department
EDLOS	Emergency department length of stay
GCS	Glasgow Coma Scale
ICD	International Classification of Diseases
ICU	Intensive care unit
IHM	In-hospital mortality
ISS	Injury Severity Score
KTAS	Korean Triage and Acuity Scale
LOS	Length of stay
MAP	Mean arterial blood pressure
MV	Mechanical ventilation
MA	Meta-analysis
MEWS	Modified Early Warning Score
NOS	Newcastle–Ottawa Quality Assessment Scale
NEWS	National Early Warning Score
NIH-SS	NIH Stroke Scale
OR	Odds ratio
PRISMA	Preferred Reporting Items for Systematic Reviews and Meta-Analyses
RTS	Revised Trauma Score
SAPS	Simplified Acute Physiology Score
SOFA	Sequential Organ Failure Assessment
TRISS	Therapeutic Intervention Scoring System
TISSS	Therapeutic Intervention Scoring System Score
