# Supplementary material for: Length-of-Stay in the Emergency Department and In-Hospital Mortality: A Systematic Review and Meta-Analysis
Source: J Clin Med. 2022 Dec 21;12(1):32. doi: 10.3390/jcm12010032 (PMC9821325; doi:10.3390/jcm12010032)
Supplement: Supplementary file 1 [file jcm-12-00032-s001.zip › Supplemental Tables.docx]

**Supplemental Table 1.** PRISMA 2009 checklist for the selection of suitable studies for inclusion in the systematic review and meta-analysis.

| **Section and Topic** | **Item #** | **Checklist item** | **Location where item is reported** |
| --- | --- | --- | --- |
| **TITLE** | | |  |
| Title | 1 | Identify the report as a systematic review. | 1 |
| **ABSTRACT** | | |  |
| Abstract | 2 | See the PRISMA 2020 for Abstracts checklist. | 4, 5 |
| **INTRODUCTION** | | |  |
| Rationale | 3 | Describe the rationale for the review in the context of existing knowledge. | 7 |
| Objectives | 4 | Provide an explicit statement of the objective(s) or question(s) the review addresses. | 7 |
| **METHODS** | | |  |
| Eligibility criteria | 5 | Specify the inclusion and exclusion criteria for the review and how studies were grouped for the syntheses. | 8 |
| Information sources | 6 | Specify all databases, registers, websites, organizations, reference lists and other sources searched or consulted to identify studies. Specify the date when each source was last searched or consulted. | 9 |
| Search strategy | 7 | Present the full search strategies for all databases, registers and websites, including any filters and limits used. | 7, 8 |
| Selection process | 8 | Specify the methods used to decide whether a study met the inclusion criteria of the review, including how many reviewers screened each record and each report retrieved, whether they worked independently, and if applicable, details of automation tools used in the process. | 8,9 |
| Data collection process | 9 | Specify the methods used to collect data from reports, including how many reviewers collected data from each report, whether they worked independently, any processes for obtaining or confirming data from study investigators, and if applicable, details of automation tools used in the process. | 9 |
| Data items | 10a | List and define all outcomes for which data were sought. Specify whether all results that were compatible with each outcome domain in each study were sought (e.g. for all measures, time points, analyses), and if not, the methods used to decide which results to collect. | 8 |
|  | 10b | List and define all other variables for which data were sought (e.g. participant and intervention characteristics, funding sources). Describe any assumptions made about any missing or unclear information. | 9 |
| Study risk of bias assessment | 11 | Specify the methods used to assess risk of bias in the included studies, including details of the tool(s) used, how many reviewers assessed each study and whether they worked independently, and if applicable, details of automation tools used in the process. | 11 |
| Effect measures | 12 | Specify for each outcome the effect measure(s) (e.g. risk ratio, mean difference) used in the synthesis or presentation of results. | 10 |
| Synthesis methods | 13a | Describe the processes used to decide which studies were eligible for each synthesis (e.g. tabulating the study intervention characteristics and comparing against the planned groups for each synthesis (item #5)). | 10 |
|  | 13b | Describe any methods required to prepare the data for presentation or synthesis, such as handling of missing summary statistics, or data conversions. | 8, 9 |
|  | 13c | Describe any methods used to tabulate or visually display results of individual studies and syntheses. | 8, 9 |
|  | 13d | Describe any methods used to synthesize results and provide a rationale for the choice(s). If meta-analysis was performed, describe the model(s), method(s) to identify the presence and extent of statistical heterogeneity, and software package(s) used. | 10, 11 |
|  | 13e | Describe any methods used to explore possible causes of heterogeneity among study results (e.g. subgroup analysis, meta-regression). | 10, 11 |
|  | 13f | Describe any sensitivity analyses conducted to assess robustness of the synthesized results. | 10, 11 |
| Reporting bias assessment | 14 | Describe any methods used to assess risk of bias due to missing results in a synthesis (arising from reporting biases). | 10 |
| Certainty assessment | 15 | Describe any methods used to assess certainty (or confidence) in the body of evidence for an outcome. | 10, 11 |
| **RESULTS** | | |  |
| Study selection | 16a | Describe the results of the search and selection process, from the number of records identified in the search to the number of studies included in the review, ideally using a flow diagram. | 11, 12 |
|  | 16b | Cite studies that might appear to meet the inclusion criteria, but which were excluded, and explain why they were excluded. | 11, 12 |
| Study characteristics | 17 | Cite each included study and present its characteristics. | 12, 13 |
| Risk of bias in studies | 18 | Present assessments of risk of bias for each included study. | 14 |
| Results of individual studies | 19 | For all outcomes, present, for each study: (a) summary statistics for each group (where appropriate) and (b) an effect estimate and its precision (e.g. confidence/credible interval), ideally using structured tables or plots. | 14, 15, 16 |
| Results of syntheses | 20a | For each synthesis, briefly summarize the characteristics and risk of bias among contributing studies. | 14, 15, 16 |
|  | 20b | Present results of all statistical syntheses conducted. If meta-analysis was done, present for each the summary estimate and its precision (e.g. confidence/credible interval) and measures of statistical heterogeneity. If comparing groups, describe the direction of the effect. | 14, 15, 16 |
|  | 20c | Present results of all investigations of possible causes of heterogeneity among study results. | 14, 15, 16 |
|  | 20d | Present results of all sensitivity analyses conducted to assess the robustness of the synthesized results. | 13, 14, 15 |
| Reporting biases | 21 | Present assessments of risk of bias due to missing results (arising from reporting biases) for each synthesis assessed. | 14 |
| Certainty of evidence | 22 | Present assessments of certainty (or confidence) in the body of evidence for each outcome assessed. | 13, 14, 15, 16 |
| **DISCUSSION** | | |  |
| Discussion | 23a | Provide a general interpretation of the results in the context of other evidence. | 16, 17 |
|  | 23b | Discuss any limitations of the evidence included in the review. | 20 |
|  | 23c | Discuss any limitations of the review processes used. | 20 |
|  | 23d | Discuss implications of the results for practice, policy, and future research. | 18 |
| **OTHER INFORMATION** | | |  |
| Registration and protocol | 24a | Provide registration information for the review, including register name and registration number, or state that the review was not registered. | 7 |
|  | 24b | Indicate where the review protocol can be accessed, or state that a protocol was not prepared. | 7 |
|  | 24c | Describe and explain any amendments to information provided at registration or in the protocol. | 7 |
| Support | 25 | Describe sources of financial or non-financial support for the review, and the role of the funders or sponsors in the review. | 3 |
| Competing interests | 26 | Declare any competing interests of review authors. | 21 |
| Availability of data, code and other materials | 27 | Report which of the following are publicly available and where they can be found: template data collection forms; data extracted from included studies; data used for all analyses; analytic code; any other materials used in the review. | 22 |

*From:* Page MJ, McKenzie JE, Bossuyt PM, Boutron I, Hoffmann TC, Mulrow CD, et al. The PRISMA 2020 statement: an updated guideline for reporting systematic reviews. BMJ 2021;372:n71. doi: 10.1136/bmj.n71

For more information, visit: <http://www.prisma-statement.org/>

**Supplemental Table 2.** Search specifications for PubMed, Medline, Embase, Web of Science, Cochrane Library, CINAHL, APA PsycInfo, and Scopus databases.

| **Source and search date** | **Search string** | **Results** | **Notes** |
| --- | --- | --- | --- |
| **PubMed**  **(NLM)**  **Coverage:**  from database inception to search date  **Search date:**  2022-01-14 | ((("Death"[MeSH] OR "Mortality"[MeSH] OR "Child Mortality"[MeSH] OR "Hospital Mortality"[MeSH] OR death*[Title/Abstract] OR mortalit*[Title/Abstract]) AND ("Emergency Responders"[MeSH] OR "Emergency Medicine"[MeSH] OR "Evidence-Based Emergency Medicine"[MeSH] OR "Emergency Service, Hospital"[MeSH] OR "Emergency Medical Services"[MeSH] OR "Emergency Services, Psychiatric"[MeSH] OR "Pediatric Emergency Medicine"[MeSH] OR "emergency room*"[Title/Abstract] OR "emergency department*"[Title/Abstract] OR "emergency ward*”[Title/Abstract] OR "emergency patient*"[Title/Abstract] OR "ED"[Title/Abstract] OR "ER"[Title/Abstract] OR "emergency medicine"[Title/Abstract] OR "emergency medical service*"[Title/Abstract] OR "emergency unit*"[Title/Abstract] OR "emergency responder*"[Title/Abstract]) AND ("Length-of-Stay"[MeSH] OR "waiting time"[Title/Abstract] OR "length-of-stay"[Title/Abstract] OR "length-of-stay"[Title/Abstract] OR "lengths of stay" [Title/Abstract] OR "LOS"[Title/Abstract] OR "wait time"[Title/Abstract] OR "EDLOS"[Title/Abstract] OR "stay length*"[Title/Abstract] OR "HLOS"[Title/Abstract] OR "IPLOS"[Title/Abstract]))) | 4,744 | All search terms are searched in the fields: Title/Abstract” and in “MeSH” when available  No filters or limitations applied |
| **Medline**  (Clarivate)  **Coverage:**  from database inception to search date  **Search date:**  2022-01-14 | (((TOPIC: "waiting time" OR "length-of-stay" OR "length-of-stay" OR "LOS" OR "wait time" OR "EDLOS" OR "stay length*" OR "IPLOS" OR "lengths of stay" OR "HLOS") AND (TOPIC: death* OR mortalit*) AND (TOPIC: "emergency room*" OR "emergency department*" OR "emergency ward*" OR "emergency patient*" OR "ED" OR "ER" OR "emergency medicine" OR "emergency medical service*" OR "emergency unit*" OR "emergency responder*"))) | **3,740** | All search terms are searched in the fields: “Topic” (including the title, abstract and author-supplied keywords)  No filters or limitations applied |
| **EMBASE** (Elsevier, all sources included)  **Coverage:**  from database inception to search date  **Search date:**  2022-01-15 | ((('emergency room*':ab,ti OR 'emergency department*':ab,ti OR 'emergency ward*':ab,ti OR 'emergency patient*':ab,ti OR 'ED':ab,ti OR 'ER':ab,ti OR 'emergency medicine':ab,ti OR 'emergency medical service*':ab,ti OR 'emergency unit*':ab,ti OR 'emergency responder*':ab,ti OR'emergency medicine'/de OR 'pediatric emergency medicine'/de OR 'psychiatric emergency service'/de OR 'evidence-based emergency medicine'/de OR 'hospital emergency service'/de OR 'rescue personnel'/de) AND ('waiting time':ab,ti OR 'length of stay':ab,ti OR 'length-of-stay':ab,ti OR 'LOS':ab,ti OR 'wait time':ab,ti OR 'EDLOS':ab,ti OR 'stay length*':ab,ti OR 'IPLOS':ab,ti OR 'lengths of stay':ab,ti OR 'HLOS':ab,ti OR 'length of stay'/de) AND 'death*':ab,ti OR 'mortalit*':ab,ti OR 'death'/de OR 'mortality'/de OR 'childhood mortality'/de OR 'hospital mortality'/de))) | **6,929** | All search terms are searched in the fields: “Title” and “Abstract” (here marked with “:ab,ti”) and in the “thesaurus” (here marked with “/de”) when available  No filters or limitations applied  Thesaurus (Emtree) variations compared to PubMed’s MeSH are applied as per availability and recommendations in Embase |
| **Web of Science** -Core collection,  Clarivate)  **Coverage:**  from database inception to search date  **Search date:**  2022-01-14 | (((TOPIC:"emergency room*" OR "emergency department*" OR "emergency ward*" OR "emergency patient*" OR “ED” OR “ER" OR “emergency medicine” OR "emergency medical service*" OR “emergency unit*” OR “emergency responder*”) AND (TOPIC: death* OR mortalit*) AND (TOPIC: "waiting time" OR "length-of-stay" OR "length-of-stay" OR “LOS” OR "wait time" OR “EDLOS” OR "stay length*" OR "IPLOS” OR “lengths of stay” OR “HLOS”))) | **2,767** | All search terms are searched in the fields: “Topic” (including the title, abstract and author-supplied keywords)  No filters or limitations applied  No thesaurus available |
| **Cochrane Library**  **(Cochrane Collaboration)**  **Coverage:**  from database inception to search date  **Search date:**  2022-01-15 | ((("Death"[MeSH] OR "Mortality"[MeSH] OR "Child Mortality"[MeSH] OR "Hospital Mortality"[MeSH] OR death*[TI/AB/KW] OR mortalit*[TI/AB/KW]) AND ("Emergency Responders"[MeSH] OR "Emergency Medicine"[MeSH] OR "Evidence-Based Emergency Medicine"[MeSH] OR "Emergency Service, Hospital"[MeSH] OR "Emergency Medical Services"[MeSH] OR "Emergency Services, Psychiatric"[MeSH] OR "Pediatric Emergency Medicine"[MeSH] OR "emergency room*"[TI/AB/KW] OR "emergency department*"[TI/AB/KW] OR "emergency ward*"[TI/AB/KW OR "emergency patient*"[TI/AB/KW] OR “ED”[ TI/AB/KW] OR “ER"[TI/AB/KW] OR “emergency medicine”[TI/AB/KW] OR "emergency medical service*"[TI/AB/KW] OR “emergency unit*”[TI/AB/KW] OR “emergency responder*”[Title/Abstract]) AND ("Length-of-Stay"[MeSH] OR "waiting time"[TI/AB/KW] OR "length-of-stay"[TI/AB/KW] OR "length-of-stay"[TI/AB/KW] OR “lengths of stay” [TI/AB/KW] OR “LOS”[TI/AB/KW] OR "wait time"[TI/AB/KW] OR “EDLOS”[TI/AB/KW] OR "stay length*"[Title/Abstract] OR “HLOS”[TI/AB/KW] OR "IPLOS”[TI/AB/KW]))) | **527** | All search terms are searched in the fields “TI/AB/KW” (including the title, abstract and author-supplied keywords) and in “MeSH” when available  **Filters applied:**  ‘trials’ |
| **CINAHL**  (EBSCOhost)  **Coverage:**  from database inception to search date  **Search date:**  2022-01-14 | (((AB ("emergency room*" OR "emergency department*" OR "emergency ward*" OR "emergency patient*" OR “ED” OR “ER" OR “emergency medicine” OR "emergency medical service*" OR “emergency unit*” OR “emergency responder*”) OR TI ("emergency room*" OR "emergency department*" OR "emergency ward*" OR "emergency patient*" OR “ED” OR “ER" OR “emergency medicine” OR "emergency medical service*" OR “emergency unit*” OR “emergency responder*”) OR MH ("Emergency Services, Psychiatric" OR "Emergency Medical Services" OR "Emergency Medicine" OR "Emergency Service") AND (TI ("waiting time" OR "length-of-stay" OR "length-of-stay" OR "LOS" OR "wait time" OR "EDLOS" OR "stay length*" OR "IPLOS" OR "lengths of stay" OR "HLOS") OR (AB "waiting time" OR "length-of-stay" OR "length-of-stay" OR "LOS" OR "wait time" OR "EDLOS" OR "stay length*" OR "IPLOS" OR "lengths of stay" OR "HLOS") OR MH ("Length-of-Stay") AND (TI (death* OR mortalit*) OR AB (death* OR mortalit*) OR MH ("Hospital Mortality" OR "Death" OR "Child Mortality" OR "Mortality"))) | **1,525** | All search terms are searched in the fields: “Title” and “Abstract” (here marked with “TI and “AB”) and in the “Subject Headings” (here marked with “MH”) when available  No filters or limitations applied.  Subject Heading variations compared to PubMed’s MeSH are applied as per availability and recommandations in CINAHL |
| **APA PsycInfo**  (EBSCO)  **Coverage:**  from database inception to search date  **Search date:**  2022-01-15 | (((TI( "emergency room*" OR "emergency department*" OR "emergency ward*" OR "emergency patient*" OR "ED" OR "ER" OR "emergency medicine" OR "emergency medical service*" OR "emergency unit*" OR "emergency responder*") OR AB ( "emergency room*" OR "emergency department*" OR "emergency ward*" OR "emergency patient*" OR "ED" OR "ER" OR "emergency medicine" OR "emergency medical service*" OR "emergency unit*" OR "emergency responder*") OR DE "Emergency Services") AND (TI ("waiting time" OR "length-of-stay" OR "length-of-stay" OR "LOS" OR "wait time" OR "EDLOS" OR "stay lengt*" OR "IPLOS" OR "lengths of stay" OR "HLOS") OR AB ("waiting time" OR "length-of-stay" OR "length-of-stay" OR "LOS" OR "wait time" OR "EDLOS" OR "stay length*" OR "IPLOS" OR "lengths of stay" OR "HLOS") AND TI (death* OR mortalit*) OR AB (death* OR mortalit*) OR DE "Death and Dying"))) | **101** | All search terms are searched in the fields: “Title” and “Abstract” (here marked with “TI and “AB”) and in the “Thesaurus” (here marked with “DE”) when available  No filters or limitations applied  Subject Heading variations compared to PubMed’s MeSH are applied as per availability and recommendations in PsycInfo |
| **Scopus**  **(Elsevier)**  **Coverage:**  from database inception to search date  **Search date:**  2020-01-14 | (((TITLE-ABS-KEY("emergency room*" OR "emergency department*" OR "emergency ward*" OR "emergency patient*" OR “ED” OR “ER" OR “emergency medicine” OR "emergency medical service*" OR “emergency unit*” OR “emergency responder*”) AND TITLE-ABS(death* OR mortalit*) AND TITLE-ABS("waiting time" OR "length-of-stay" OR "length-of-stay" OR “LOS” OR "wait time" OR “EDLOS” OR "stay length*" OR "IPLOS” OR “lengths of stay” OR “HLOS”))) | **2,843** | All search terms are searched in the fields: including:“title”, and “abstract” (here marked with “TITLE-ABS“ alternatively “title”, “abstract”, “keywords” (here marked with “TITLE-ABS-KEY“  No thesaurus available.  No filters or limitations applied |
| Total number of records identified: | | **23,176** | |
| Total number of unique records after de-duplication: | | **11,337** | |

**Supplemental Table 3. Quality assessment of the studies.** The Newcastle-Ottawa Quality Assessment Scale consists of four items on study selection, one item on comparability and three items on study outcomes (see reference 20 in main manuscript). According to this scale, studies can be awarded one star for each of the four items on selection and for each of the 3 items on outcomes and a maximum of two stars for comparability. Stars are awarded such that the highest quality studies are awarded up to nine stars. The evaluation was performed by two independent evaluators (E1 and E2). The average is 6.53 ± 1.23 (Min: 3 and Max: 8).

| **Reference** | **Selection** | | | | **Comparability of cohorts** | **Outcome** | | | **Total Score** |
| --- | --- | --- | --- | --- | --- | --- | --- | --- | --- |
|  | **Representativeness of the exposed cohort** | **Selection of the non-exposed cohort** | **Ascertainment of exposure** | **Outcome of interest not present at the study start** |  | **Assessment** | **Length of follow-up** | **Adequacy of follow-up of the cohorts** |  |
| Carter et al. (27)  E1  E2 | *  * | *  * | *  * | *  * | *  * | *  * | *  * | *  * | 8  8 |
| Mitra et al. (28)  E1  E2 |  | *  * | *  * | *  * |  | *  * | *  * | *  * | 6  6 |
| Chong et al. (29)  E1  E2 | *  * | *  * | *  * | *  * | *  * | *  * | *  * | *  * | 8  8 |
| Flabouris et al. (30)  E1  E2 | *  * | *  * | *  * | *  * |  | *  * | *  * | *  * | 7  7 |
| Akhtar et al. (31)  E1  E2 | *  * | *  * | *  * | *  * |  | *  * | *  * | *  * | 7  7 |
| Dierks et al. (32)  E1  E2 | *  * | *  * | *  * | *  * |  | *  * | *  * | *  * | 7  7 |
| Chen et al. (33)  E1  E2 | *  * | *  * | *  * | *  * |  | *  * | *  * | *  * | 7  7 |
| Jones et al. (34)  E1  E2 | *  * | *  * |  | *  * | *  * | *  * |  | *  * | 6  6 |
| Mowery et al. (35)  E1  E2 | *  * | *  * | *  * | *  * | *  * | *  * | *  * | *  * | 8  8 |
| Serviá et al. (36)  E1  E2 | *  * | *  * | *  * |  | *  * | *  * | *  * | *  * | 7  7 |
| Tilluckdharry et al. (37)  E1  E2 | *  * | *  * | *  * | *  * | *  * | *  * | *  * | *  * | 8  8 |
| Hirshi et al. (38)  E1  E2 | *  * | *  * | *  * | *  * | *  * | *  * | *  * | *  * | 8  8 |
| Plunkett et al. (39)  E1  E2 | *  * |  | *  * | *  * |  | *  * | *  * | *  * | 6  6 |
| García-Gigorro et al. (40)  E1  E2 | *  * | * | *  * | *  * | *  * | *  * |  |  | 5  6 |
| Agustin et al. (41)  E1  E2 | *  * | *  * | *  * | *  * |  | *  * |  |  | 5  5 |
| Siletz et al. (42)  E1  E2 | *  * | *  * | *  * | *  * | *  * | *  * |  |  | 6  6 |
| Junhasavasdikul et al. (43)  E1  E2 | * | * | *  * | *  * |  | *  * |  |  | 3  5 |
| Soni et al. (44)  E1  E2 | * |  | *  * | *  * |  | *  * |  |  | 3  4 |
| Paton et al. (45)  E1  E2 | *  * | *  * | *  * | *  * | *  * | *  * |  |  | 6  6 |
| Zhang et al. (46)  E1  E2 | *  * | *  * | *  * | *  * | *  * | *  * |  |  | 6  6 |
| Intas et al. (47)  E1  E2 | *  * | *  * | *  * | *  * | *  * | *  * |  |  | 6  6 |
| Richardson et al. (41)  E1  E2 | *  * | *  * | *  * | *  * | *  * | *  * |  |  | 6  6 |
| Aitavaara‐Anttila et al. (48)  E1  E2 | *  * | *  * | *  * | *  * | *  * | *  * | *  * | *  * | 8  8 |
| Khan et al. (49)  E1  E2 | *  * | *  * | *  * | *  * | *  * | *  * |  |  | 6  6 |
| Byrne et al. (50)  E1  E2 | *  * | *  * | *  * | *  * | *  * | *  * | *  * | *  * | 8  8 |
| Groenland et al. (51)  E1  E2 | *  * | *  * | *  * | *  * | *  * | *  * | *  * | *  * | 8  8 |
| Haji et al. (52)  E1  E2 | *  * | *  * | *  * | *  * | *  * | *  * |  |  | 6  6 |
| Ribeiro et al. (53)  E1  E2 | *  * | *  * | *  * | *  * | *  * | *  * |  |  | 6  6 |
| Lin et al. (54)  E1  E2 | *  * | *  * | *  * | *  * | *  * | *  * |  |  | 6  6 |
| Mejaddam et al. (55)  E1  E2 | *  * | *  * | *  * | *  * | *  * | *  * |  |  | 6  6 |
| Saukonnen et al. (56)  E1  E2 | *  * | *  * | *  * | *  * | *  * | *  * |  |  | 6  6 |
| Hung et al. (57)  E1  E2 | *  * | *  * | *  * | *  * | *  * | *  * |  |  | 6  6 |
| Cardoso et al. (58)  E1  E2 | *  * | *  * | *  * | *  * | *  * | *  * |  |  | 6  6 |
| Ashkenazi (59)  E1  E2 | *  * | *  * | *  * | *  * | *  * | *  * | *  * | *  * | 8  8 |
| Stey (60)  E1  E2 | *  * | *  * | *  * | *  * | * | *  * | *  * | * | 7  7 |
| Choi (61)  E1  E2 | *  * | *  * | *  * | *  * | *  * | *  * |  | * | 7  6 |
| Lin (62)  E1  E2 | *  * | *  * | *  * | *  * | *  * | *  * |  | * | 7  6 |
| Altreby (63)  E1  E2 | *  * | *  * | *  * | *  * | *  * | * | * | * | 7  6 |
| Wessman (64)  E1  E2 | *  * | *  * | *  * | *  * | * | *  * | *  * | *  * | 8  7 |
| Rana (65)  E1  E2 | *  * | * | *  * | *  * |  | *  * | *  * |  | 5  6 |
| Thibon (66)  E1  E2 | * |  | *  * | *  * | *  * | *  * | *  * | * * | 6  7 |
| Asheim (67)  E1  E2 | *  * |  | *  * | *  * | *  * | *  * | *  * | *  * | 7  7 |
| Cheng (68)  E1  E2 | *  * |  | *  * | *  * | *  * | *  * | *  * | *  * | 6  7 |
| Crilly (69)  E1  E2 | *  * |  | *  * | *  * |  | *  * | *  * | * | 5  6 |
| Davis (70)  E1  E2 | *  * | *  * | *  * | *  * | *  * | *  * | *  * | *  * | 8  8 |
| Elay (71)  E1  E2 |  |  | *  * | *  * |  | *  * | *  * | *  * | 5  5 |
| Rose (72)  E1  E2 | *  * | *  * |  | *  * | *  * | *  * | *  * | *  * | 7  7 |
| Sabaz (73)  E1  E2 | * | * | * | *  * | *  * | *  * | *  * | *  * | 5  8 |
| Verma (74)  E1  E2 | *  * | * | *  * | *  * |  | *  * | *  * | * | 5  7 |
| Derose (10)  E1  E2 |  |  |  |  |  |  |  |  |  |
| Jain (75)  E1  E2 | *  * | *  * | *  * | *  * | *  * | *  * | *  * | *  * | 8  8 |
| Total NOS Score |  |  |  |  |  |  |  |  | Mean=6.53  ±1.23  Max=8  Min=3 |

**Supplemental Table 4.** Summary of the random-effects model results (DerSimonian-Laird).

| **Cut-off** | **Experiment** | **N.** | **Effect size** | **CI (95%)** | | **p-v*** | **Heterogeneity test** | | |
| --- | --- | --- | --- | --- | --- | --- | --- | --- | --- |
|  |  |  |  | **LB** | **UB** |  | **Cochran Q** | **Het p-v** | $\boldsymbol{I}^{\boldsymbol{2}}$ |
| Low EDLOS | All studies | 9 | 0.954 | 0.685 | 1.330 | 0.783 | 32.628 | <0.001 | 75.481% |
|  | ICU | 7 | 1.121 | 0.728 | 1.726 | 0.605 | 23.437 | <0.001 | 74.4% |
|  | Non-ICU | 2 | 0.581 | 0.453 | 0.745 | <0.001 | 0.814 | 0.367 | 0% |
| 4 hours | All studies | 7 | 0.958 | 0.455 | 2.018 | 0.910 | 221.461 | <0.001 | 97.29% |
|  | ICU | 4 | 1.379 | 0.581 | 3.272 | 0.466 | 25.302 | <0.001 | 88.14% |
|  | Non-ICU | 3 | 0.689 | 0.272 | 1.747 | 0.432 | 81.918 | <0.001 | 97.56% |
| 5 hours | All studies | 6 | 1.005 | 0.494 | 2.046 | 0.989 | 26.539 | <0.001 | 81.16% |
|  | ICU | 5 | 1.360 | 0.741 | 2.496 | 0.320 | 12.476 | 0.014 | 67.94% |
|  | Non-ICU | 1 | 0.332 | 0.162 | 0.680 | * | * | * | * |
| 6 hours | All studies | 14 | 0.952 | 0.690 | 1.315 | 0.766 | 450.284 | <0.001 | 97.11% |
|  | ICU | 12 | 1.020 | 0.648 | 1.606 | 0.931 | 394.057 | <0.001 | 97.21% |
|  | Non-ICU | 2 | 0.606 | 0.311 | 1.178 | 0.140 | 3.737 | 0.053 | 73.24% |
| 8 hours | All studies | 12 | 1.064 | 0.838 | 1.352 | 0.611 | 212.188 | <0.001 | 94.82% |
|  | ICU | 5 | 1.036 | 0.751 | 1.429 | 0.829 | 29.699 | <0.001 | 86.53% |
|  | Non-ICU | 7 | 1.080 | 0.706 | 1.653 | 0.723 | 181.683 | <0.001 | 96.7% |
| 24 hours | All studies | 7 | 1.220 | 0.851 | 1.748 | 0.279 | 11.025 | 0.088 | 45.58% |
|  | ICU | 6 | 1.396 | 1.147 | 1.701 | <0.001 | 4.181 | 0.524 | 0% |
|  | Non-ICU | 1 | 0.272 | 0.081 | 0.912 | * | * | * | * |

* Impossible to prepare full MA because of the single study

**Supplemental Table 5.** Publication bias tests for all cut-offs.

| Cut-off | **Egger's test** | | | **Begg's test** | | |
| --- | --- | --- | --- | --- | --- | --- |
|  | Intercept, CI (95%) | p-value | Kendall's Tau | | p-value |  |
| Low EDLOS | 0.3946 (-1.7990,2.5882) | 0.6833 | 0.0000 | | 1.0000 |  |
| 4 hours | 0.3475 (-11.0962,11.7913) | 0.9408 | -0.3333 | | 0.2931 |  |
| 5 hours | -3.0013 (-11.8955,5.8928) | 0.4019 | -0.0667 | | 0.8510 |  |
| 6 hours | 0.9347 (-3.8969,5.7663) | 0.6808 | -0.2967 | | 0.1394 |  |
| 8 hours | 2.5464 (-1.2796,6.3724) | 0.1689 | -0.1212 | | 0.5833 |  |
| 24 hours* | -1.9685 (-6.2241,2.2870) | 0.2374 | -0.6000 | | 0.1416 |  |

* Impossible to produce MA since in two of seven studies there were no dead cases in intervention group. Egger's test and Begg's test for the rest of the five studies.

**Supplemental Table 6.** Publication bias tests for all cut-offs in the ICU population.

| Cut-off | **Egger's test** | | | **Begg's test** | | |
| --- | --- | --- | --- | --- | --- | --- |
|  | Intercept, CI (95%) | p-value | Kendall's Tau | | p-value |  |
| Low EDLOS | 0.9990 (-1.4405,3.4385) | 0.3407 | 0.1429 | | 0.6523 |  |
| 4 hours | -3.7757 (-10.3186,2.7373) | 0.1311 | -0.6667 | | 0.1742 |  |
| 5 hours | -2.6001 (-10.1598,4.9596) | 0.3537 | -0.2000 | | 0.6242 |  |
| 6 hours | -0.2066 (-6.5650,6.1517) | 0.9437 | -0.2121 | | 0.3337 |  |
| 8 hours | 1.5535 (-3.8091,6.9161) | 0.4246 | -0.2000 | | 0.6242 |  |
| 24 hours* | -0.6835 (-7.6443,6.2774) | 0.7138 | -0.3333 | | 0.4969 |  |

* Impossible to produce MA since in two of six studies there were no dead cases in intervention group. Egger's test and Begg's test for the rest of the four studies.

**Supplemental Table 7.** Publication bias tests for all cut-offs in the non-ICU population.

| Cut-off | **Egger's test** | | **Begg's test** | |
| --- | --- | --- | --- | --- |
|  | Intercept, CI (95%) | p-value | Kendall's Tau | p-value |
| Low EDLOS | -1.011* | P<0.001 | -1.0000 | 0.3173 |
| 4 hours | 0.03015 (-148.960,149.020) | 0.9984 | 0.3333 | 0.6015 |
| 6 hours | -2.0971* | P<0.001 | -1.0000 | 0.3173 |
| 8 hours | 3.5446 (-4.2344,11.3235) | 0.2942 | -0.04762 | 0.8806 |

* Unable to calculate CI for the group with two studies

**Supplemental Table 8.** Sensitivity analysis (leave-one-out) results of the overall data. Random-effects model (DerSimonian-Laird).

| **Cut-off** | **Study** | $\boldsymbol{I}^{\boldsymbol{2}}$ | **Cochran Q** | **Overall estimated effect for the rest**  **CI 95%** | **p-v** |
| --- | --- | --- | --- | --- | --- |
| Low EDLOS  $I^{2}=75.481\%$ | Aitavaara-Anttilla | 77.946% | 31.741 | 0.936 (0.650,1.347) | 0.720 |
|  | Davis | 71.894% | 24.906 | 1.068 (0.696,1.638) | 0.764 |
|  | Groenland | 78.542% | 32.622 | 0.981 (0.575,1.674) | 0.945 |
|  | Intas | 76.100% | 29.288 | 0.897 (0.633,1.270) | 0.539 |
|  | Juhanavasdikul | 77.594% | 31.241 | 0.981 (0.703,1.370) | 0.911 |
|  | Mowery | 66.866% | 21.126 | 0.836 (0.611,1.145) | 0.265 |
|  | Servia | 77.865% | 31.624 | 1.022 (0.709,1.473) | 0.908 |
|  | Siletz | 74.147% | 27.076 | 0.883 (0.640,1.217) | 0.447 |
|  | Soni | 76.712% | 30.059 | 0.990 (0.713,1.375) | 0.952 |
| 4 hours  $\boldsymbol{I}^{\boldsymbol{2}}$=97.302% | Altreby | 95.162% | 103.338 | 0.786 (0.392,1.579) | 0.499 |
|  | Ashkenazi | 90.975% | 55.405 | 1.239 (0.699,2.195) | 0.464 |
|  | Intas | 97.651% | 212.866 | 0.801 (0.354,1.809) | 0.593 |
|  | Juhanavasdikul | 97.710% | 218.304 | 1.072 (0.477,2.410) | 0.867 |
|  | Paton | 97.598% | 208.182 | 0.843 (0.305,2.329) | 0.741 |
|  | Serviá | 97.696% | 217.007 | 1.152 (0.534,2.486) | 0.719 |
|  | Soni | 97.747% | 221.931 | 0.969 (0.423,2.221) | 0.941 |
| 5 hours  $\boldsymbol{I}^{\boldsymbol{2}}$=81.159% | Garcia-Gigorro | 78.942% | 18.995 | 0.802 (0.380,1.694) | 0.563 |
|  | Intas | 80.611% | 20.631 | 0.831 (0.366,1.889) | 0.658 |
|  | Jones | 84.864% | 26.427 | 0.977 (0.410,2.326) | 0.958 |
|  | Juhanavasdikul | 67.939% | 12.476 | 0.360 (0.741,2.496) | 0.320 |
|  | Serviá | 82.493% | 22.848 | 1.175 (0.578,2.390) | 0.656 |
|  | Soni | 84.676% | 26.103 | 0.974 (0.387,2.453) | 0.955 |
| 6 hours  $\boldsymbol{I}^{\boldsymbol{2}}$=97.113% | Augustin | 97.331% | 449.588 | 0.940 (0.672,1.317) | 0.720 |
|  | Badar | 97.315% | 446.867 | 0.922 (0.659,1.291) | 0.637 |
|  | Byrne | 97.013% | 401.691 | 0.952 (0.614,1.474) | 0.824 |
|  | Choi | 97.324% | 448.402 | 0.934 (0.666,1.311) | 0.695 |
|  | Intas | 97.300% | 444.443 | 0.909 (0.651,1.270) | 0.577 |
|  | Juhanavasdikul | 97.304% | 445.070 | 1.009 (0.724,1.408) | 0.956 |
|  | Lin | 97.278% | 440.901 | 0.935 (0.627,1.395) | 0.742 |
|  | Rana | 95.691% | 278.477 | 0.814 (0.616,1.076) | 0.148 |
|  | Ree Ann Hirshi | 97.335% | 450.242 | 0.961 (0.688,1.343) | 0.815 |
|  | Richardson | 95.064% | 243.114 | 1.183 (0.910,1.539) | 0.209 |
|  | Servia | 97.325% | 448.626 | 0.979 (0.707,1.356) | 0.899 |
|  | Soni | 97.333% | 449.952 | 0.969 (0.692,1.355) | 0.853 |
|  | Stey | 97.248% | 436.003 | 0.933 (0.633,1.374) | 0.724 |
|  | Zhongheng | 97.210% | 430.119 | 0.916 (0.650,1.291) | 0.617 |
| 8 hours  $\boldsymbol{I}^{\boldsymbol{2}}$=94.812% | Akhtar | 95.102% | 204.168 | 1.014 (0.797,1.290) | 0.908 |
|  | Carter | 95.206% | 208.577 | 1.111 (0.804,0.537) | 0.524 |
|  | Chen | 95.283% | 212.010 | 1.070 (0.840,1.363) | 0.583 |
|  | Chong | 90.984% | 110.915 | 0.940 (0.771,1.145) | 0.537 |
|  | Diercks | 94.907% | 196.352 | 1.068 (0.817,1.396) | 0.630 |
|  | Flabouris | 92.678% | 136.576 | 1.146 (0.878,1.495) | 0.315 |
|  | Intas | 95.042% | 201.679 | 1.008 (0.789,1.287) | 0.950 |
|  | Juhanavasdikul | 95.179% | 207.422 | 1.136 (0.889,1.452) | 0.309 |
|  | Mitra | 95.110% | 204.508 | 1.069 (0.822,1.390) | 0.619 |
|  | Servia | 95.272% | 211.523 | 1.073 (0.844,1.365) | 0.565 |
|  | Soni | 95.283% | 212.013 | 1.083 (0.845,1.388) | 0.530 |
|  | Verma | 94.828% | 193.350 | 1.057 (0.815,1.372) | 0.676 |
| 24 hours  $\boldsymbol{I}^{\boldsymbol{2}}$=45.578% | Choi | 41.038% | 8.480 | 1.059 (0.696,1.610) | 0.789 |
|  | Intas | 53.233% | 10.691 | 1.228 (0.809,1.865) | 0.335 |
|  | Juhanavasdikul | 0% | 4.181 | 1.396 (1.147,1.701) | <0.001 |
|  | Servia | 53.428% | 10.736 | 1.198 (0.822,1.746) | 0.348 |
|  | Soni | 54.242% | 10.927 | 1.200 (0.820,1.757) | 0.347 |
|  | Tillukdharry | 47.339% | 9.495 | 1.263 (0.804,1.982) | 0.210 |
|  | Verma | 52.229% | 10.467 | 1.078 (0.615,1.889) | 0.794 |

**Supplemental Table 9.** Sensitivity analysis (leave-one-out) results of the ICU population. Random-effects model (DerSimonian-Laird).

| **Cut-off** | **Study** | $\boldsymbol{I}^{\boldsymbol{2}}$ | **Cochran Q** | **Overall estimated effect for the rest**  **CI 95%** | **p-v** |
| --- | --- | --- | --- | --- | --- |
| Low EDLOS  $I^{2}$=74.400% | Aitavaara-Anttila | 78.054% | 22.783 | 1.121 (0.674,1.865) | 0.660 |
|  | Groenland | 66.678% | 15.005 | 1.222 (0.703,2.124) | 0.478 |
|  | Intas | 75.650% | 20.534 | 1.057 (0.656,1.704) | 0.820 |
|  | Mowery | 61.827% | 13.098 | 0.988 (0.646,1.510) | 0.954 |
|  | Serviá | 77.406% | 22.130 | 1.265 (0.761,2.101) | 0.364 |
|  | Siletz | 72.582% | 18.236 | 1.015 (0.668,1.543) | 0.944 |
|  | Soni | 75.896% | 20.744 | 1.197 (0.782,1.830) | 0.408 |
| 4 hours  $I^{2}=88.143\%$ | Altreby | 85.191% | 13.505 | 0.857 (0.242,3.035) | 0.811 |
|  | Intas | 92.095% | 25.300 | 0.909 (0.234,3.530) | 0.890 |
|  | Serviá | 86.713% | 15.052 | 1.986 (0.948,4.160) | 0.069 |
|  | Soni | 82.364% | 11.340 | 1.789 (0.726,4.406) | 0.206 |
| 5 hours  $I^{2}=67.939\%$ | Garcia-Gigorro | 63.568% | 8.235 | 1.109 (0.578,2.127) | 0.756 |
|  | Intas | 70.519% | 10.176 | 1.135 (0.521,2.472) | 0.750 |
|  | Jones | 73.731% | 11.420 | 1.424 (0.668,3.038) | 0.360 |
|  | Serviá | 61.476% | 7.787 | 1.593 (0.941,2.698) | 0.083 |
|  | Soni | 68.696% | 9.583 | 1.474 (0.691,3.144) | 0.316 |
| 6 hours  $I^{2}$=97.209% | Augustin | 97.462% | 394.021 | 1.011 (0.625,1.633) | 0.966 |
|  | Badar | 97.454% | 392.700 | 0.987 (0.611,1.596) | 0.958 |
|  | Choi | 97.460% | 393.776 | 1.004 (0.618,1.630) | 0.988 |
|  | Intas | 97.441% | 390.817 | 0.969 (0.601,1.562) | 0.898 |
|  | Lin | 97.458% | 393.336 | 1.000 (0.535,1.868) | 0.999 |
|  | Rana | 95.882% | 248.878 | 0.859 (0.575,1.283) | 0.457 |
|  | Ree Ann Hirshi | 97.459% | 393.551 | 1.038 (0.645,1.671) | 0.878 |
|  | Richardson | 93.010% | 143.056 | 1.337 (0.977,1.827) | 0.069 |
|  | Serviá | 97.448% | 391.925 | 1.072 (0.676,1.700) | 0.767 |
|  | Soni | 97.454% | 392.704 | 1.049 (0.650,1.690) | 0.846 |
|  | Stey | 97.462% | 393.936 | 0.996 (0.537,1.846) | 0.989 |
|  | Zhongheng | 97.407% | 385.707 | 0.981 (0.590,1.630) | 0.940 |
| 8 hours  $I^{2}$=86.531% | Carter | 51.321% | 6.163 | 1.204 (0.795,1.823) | 0.829 |
|  | Intas | 84.796% | 19.731 | 0.904 (0.669,1.222) | 0.512 |
|  | Serviá | 89.723% | 29.190 | 1.053 (0.760,1.459) | 0.757 |
|  | Soni | 89.897% | 29.695 | 1.079 (0.754,1.543) | 0.678 |
|  | Verma | 74.291% | 11.669 | 1.023 (0.570,1.835) | 0.939 |
| 24 hours  $I^{2}$=0% | Choi | 0% | 2.183 | 1.312 (1.057,1.627) | 0.014 |
|  | Intas | 0% | 3.772 | 1.419 (1.159,1.738) | <0.001 |
|  | Serviá | 0% | 3.914 | 1.393 (1.143,1.697) | <0.001 |
|  | Soni | 2.344% | 4.096 | 1.393 (1.135,1.709) | 0.001 |
|  | Tilluckhdarry | 0% | 2.158 | 1.486 (1.199,1.842) | <0.001 |
|  | Verma | 3.163% | 4.131 | 1.355 (0.972,1.889) | 0.073 |

**Supplemental Table 10.** Sensitivity analysis (leave-one-out) results of the non-ICU population. Random-effects model (DerSimonian-Laird).

| **Cut-off** | **Study** | $\boldsymbol{I}^{\boldsymbol{2}}$ | **Cochran Q** | **Overall estimated effect for the rest CI 95%** | **p-v** |
| --- | --- | --- | --- | --- | --- |
| 4 hours  $I^{2}$=97.559% | Ashkenazi | 86.122% | 7.206 | 0.882 (0.299,2.605) | 0.820 |
|  | Juhanavasdikul | 98.761% | 80.689 | 0.802 (0.256,2.510) | 0.705 |
|  | Paton | 0% | 0.015 | 0.450 (0.382,0.529) | <0.001 |
| 8 hours  $I^{2}$=96.694% | Akhtar | 97.118% | 173.468 | 0.959 (0.618,1.487) | 0.852 |
|  | Chen | 97.245% | 181.505 | 1.098 (0.708,1.701) | 0.676 |
|  | Chong | 93.225% | 73.798 | 0.883 (0.626,1.245) | 0.478 |
|  | Diercks | 96.902% | 161.397 | 1.092 (0.610,1.956) | 0.766 |
|  | Flabouris | 92.224% | 64.297 | 1.224 (0.793,1.888) | 0.362 |
|  | Juhanavasdikul | 97.177% | 177.099 | 1.252 (0.796,1.969) | 0.331 |
|  | Mitra | 97.099% | 172.335 | 1.092 (0.647,1.845) | 0.741 |

Comment: the sensitivity analysis for non-ICU subgroup for the rest cut-offs is impossible because of the small number of studies in the subgroup (1/2).

**Supplemental Table 11.** Heterogeneity analysis: comparison between inverse variance (IV) and inverse variance heterogeneity (IVHet) methods. Estimated effect size and CI (95%).

| **Cut-off** | **Method** | **All studies** | **ICU** | **Non-ICU** |
| --- | --- | --- | --- | --- |
| Low EDLOS | IV method | 0.821 (0.755,0.893) | 0.859 (0.785,0.939) | 0.581 (0.453,0.745) |
|  | IVHet method | 0.821 (0.458,1.471) | 0.859 (0.385,1.916) | 0.581 (0.453,0.745) |
| 4 h | IV method | 1.056 (0.951,1.173) | 2.593 (2.133,3.153) | 0.734 (0.648,0.831) |
|  | IVHet method | 1.056 (0.385,2.901) | 2.593 (0.769,8.740) | 0.734 (0.248,2.171) |
| 5 h | IV method | 1.166 (0.872,1.560) | 1.491 (1.085,2.049) | * |
|  | IVHet method | 1.166 (0.554,2.453) | 1.491 (0.797,2.792) | * |
| 6 h | IV method | 0.888 (0.852,0.925) | 1.063 (0.997,1.133) | 0.782 (0.741,0.825) |
|  | IVHet method | 0.888 (0.449,1.755) | 1.063 (0.486,2.322) | 0.782 (0.340,1.796) |
| 8 h | IV method | 0.823 (0.791,0.857) | 0.839 (0.792,0.889) | 0.809 (0.766,0.855) |
|  | IVHet method | 0.823 (0.577,1.174) | 0.839 (0.532,1.323) | 0.809 (0.421,1.555) |
| 24 h | IV method | 1.339 (1.102,1.626) | 1.396 (1.147,1.701) | * |
|  | IVHet method | 1.339 (0.882,2.032) | 1.396 (1.147,1.701) | * |

* Impossible to provide the results because of the single study in the subgroup

**Supplemental Table 12:** Meta-regression analysis^*^. Discrete (categorical) factors are population type, country, and number of patients included in the studies. The continuous factors were age and sex.

Discrete (categorical) factors = population type, severity (ICU/not ICU), country, disease.

Continuous factors = age and % male.

| Cut-off | Factor | coefficient | CI (95%) | Std. error | p-v | Omnibus p-v |
| --- | --- | --- | --- | --- | --- | --- |
| Low EDLOS | **Intercept**  **Population type**  *Specific (8)*  *General (1)*  **Severity score**  *ICU (7)*  *Non-ICU (2)*  **Decease**  *Critical (2)*  *General (3)*  *Trauma (4)* | -0.195  -1.124  -0.988  0.652  -1.234 | -0.289, -0.101  -3.566,1.317  -1.737,-0.239  -5.109,-0.454  -2.008,-0.460 | 0.048  1.246  0.382  1.187  0.395 | <0.001  0.367  0.010  0.019  0.002 | 0.000 |
| 4 hours | **Intercept**  **Population type**  *General (4)*  *Specific (3)*  **Severity score**  *ICU (4)*  *Non-ICU (3)*  **Decease**  *Critical (1)*  *General (4)*  *Trauma (2)* | 1.136  -1.671  -2.972  1.502  NA | 0.302, 1.969  -2.908, -0.433  -4.643, -1.302  0.150,2.855  NA | 0.425  0.631  0.853  0.690  NA | 0.008  0.008  <0.001  0.029  NA | 0.004 |
| 5 hours | **Intercept**  **Population type**  *Specific (5)*  *General (1)*  **Severity score**  *ICU (5)*  *Non-ICU (1)*  **Decease**  *Critical patients (1)*  *General (2)*  *Stroke (1)*  *Trauma (2)* | 1.084  -1.877  -0.311  -1.052  -1.228  NA | 0.359, 1.809  -2.799, -0.955  -1.238, 0.616  -2.109, 0.005  -2.138, -0.319  NA | 0.370  0.471  0.473  0.539  0.464  NA | 0.003  <0.001  0.511  0.051  0.008  NA | 0.000 |
| 6 hours | **Intercept**  **Population type**  *Specific (9)*  *General (2)*  **Severity score**  *ICU (12)*  *Non-ICU (2)*  **Decease**  *Sepsis (3)*  *Critical patients (5)*  *General (3)*  *Trauma (3*) | -0.315  0.841  -1.661  -0.429  0.892  -1.919 | -0.672, 0.043  0.459, 1.223  -2.346, -0.976  -0.655, -0.204  0.223, 1.562  -2.380, -1.458 | 0.182  0.195  0.350  0.115  0.342  0.235 | 0.084  <0.001  <0.001  <0.001  0.009  <0.001 | 0.001 |
| 8 hours | **Intercept**  **Population type**  *Specific (7)*  *General (5)*  **Severity score**  *ICU (5)*  *Non-ICU (7)*  **Decease**  *Stroke (1)*  *Coronary syndr. (2)*  *General (6)*  *Trauma (2)*  *Critical (1)* | -0.242  1.175  1.558  -0.986  -1.273  -0.155  0.984  0.499 | -0.884, 0.401  -2.458, 0.109  0.174, 2.942  -4.018,2.046  -2.691, 0.115  -3.390, 3.080  -0.088, 2.056  -2.987,3.985 | 0.328  0.655  0.706  1.547  0.708  1.651  0.547  1.779 | 0.461  0.073  0.027  0.524  0.072  0.925  0.072  0.779 | 0.526 |
| 24 hours | **Intercept**  **Population type**  *Specific (4)*  *General (1)*  **Severity score**  *ICU (6)*  *Non-ICU (1)*  **Decease**  *Critical patients (1)*  *General (2)*  *Trauma (2)* | 1.143  -1.348  -1.139  -1.096  NA | -1.637,3.923  -2.828, 0.133  -1.030, 0.944  -4.005, 1.812  NA | 1.418  0.755  0.504  1.484  NA | 0.420  0.074  0.932  0.460  NA | 0.184 |

* The number of covariates depends on degrees of freedom in the analysis. In meta-regression analysis for five studies the maximal number of covariates that could be used was three. Therefore, the best model for the existing covariates is provided.
