# Supplementary material for: Length-of-Stay in the Emergency Department and In-Hospital Mortality: A Systematic Review and Meta-Analysis
Source: J Clin Med. 2022 Dec 21;12(1):32. doi: 10.3390/jcm12010032 (PMC9821325; doi:10.3390/jcm12010032)
Supplement: Supplementary file 1 [file jcm-12-00032-s001.zip › jcm-2041231-supplementary-Text.docx]

**Supplemental Text**

***Non-intensive care unit-admitted population***

Derose et al. found that after adjusting the analysis for confounding factors, EDLOS was not associated with IHM [9]. Mitra et al. found that prolonged (>8 h) EDLOS was associated with significantly higher IHM only when ED management care was completed in <4 h according to the 4 h-golden hours [26]. In contrast, Chong et al. found that EDLOS, regardless of whether it was prolonged (>8 h) or short (<4 h), was not associated with an increased IHM [27]. Flabouris et al. observed an association between EDLOS and IHM in patients admitted to the general ward; however, when considering confounding factors, such as triage category, gender, and admission source, EDLOS was not deemed to be a predictor of IHM [28]. Akhtar et al. found that an EDLOS <8 h was associated with a lower IHM among acute stroke patients [29]. Diercks et al*.* observed that, among patients with a non-ST elevation myocardial infarction, a longer EDLOS was associated with a higher risk of recurrent acute myocardial infarction, but not with IHM [30]. Similarly, Chen et al. found no statistically significant differences in IHM among patients with prolonged (>8 h) and intermediate (<8 h) EDLOS [31]. Among trauma patients, Mowery et al. found that the IHM increased with each hourly increment in EDLOS [33].

Plunkett et al. observed that EDLOS (as an amalgamation of door-to-team evaluation and team-to-ward times) was a significant predictor of mortality within 30 days, particularly if EDLOS was >4 h [37]. Junhasavasdikul et al. found no significant association between EDLOS and IHM [41]. Paton et al. observed that a shorter EDLOS was associated with a reduced IHM among adults admitted to the general ward, specifically among adults with an EDLOS between 3.5 and 4 h [43]. Richardson et al. analyzed all trauma patients admitted to an ED Level I trauma center divided into a non-delayed (6 h) group or a delayed (6 h) group [46]. Mortality did not increase with time spent in the ED, but decreased with an EDLOS >4 h [46]. Byrne et al*.* observed that an increase in the median EDLOS by 50% was associated with a decline in 30-day IHM from 6.2% to 4.9%, and shorter EDLOS (<4 h) was associated with a higher 30-day IHM among patients admitted to the general ward [49]. Ashkenazi et al. did not find an increased IHM in patients with EDLOS beyond 4 h and found that EDLOS was shorter in patients who died in hospital [57]. Older age was associated with IHM.

Wessman et al. found a relationship between EDLOS and IHM according to the triage priority level [62]. Surprisingly, a positive association was observed between EDLOS and 7- and 30-day mortality rate in patients with lower medical urgency (triage severity levels 2 to 4) and patients not admitted to in-hospital care. In contrast, this was not observed in patients with high medical urgency (triage priority level 1) and in patients admitted to in-hospital care. Thibon et al. found mortality rates was higher when patients wait in the emergency medicine department due to lack of bed in ward [ 64].

Asheim et al. did not find an association between EDLOS and IHM [65]. No influence of prioritization was observed, with no significant effect of EDLOS on patient safety. Cheng et al. observed an increase of poor patient outcomes when EDLOS increased [66]. The mortality increased when EDLOS exceeded 24 h [66].

Davis et al. found that female sex, more severe stroke on presentation, higher number of co-morbidities, and increased EDLOS were associated with IHM. Patients with low EDLOS had low IHM [68]. Patients with an EDLOS ≥3 h had a 37% increase in odds for IHM [69]. In Davis et al.’s study, age, sex, race, and co-morbidities were not significant predictors of EDLOS and IHM [68]. Jain et al. also did not find an association between EDLOS and IHM [72].

***Intensive care unit-admitted emergency department population***

Carter et al*.* were unable to demonstrate an adverse relationship between EDLOS and IHM in ICU patients [25]. Jones et al. demonstrated that an EDLOS >5 h was a significant predictor of poor functional outcome and discharge NIH-SSS, but not discharge disposition or death [32]. Serviá et al. showed that EDLOS was not independently associated with IHM, noting that severe trauma was associated with a reduced EDLOS but with increased IHM [34]. Tilluckdharry et al. demonstrated a mortality equivalence between those who had an EDLOS >24 h or <24 h [35]. Hirschy et al. found no statistically significant differences in IHM among septic patients with an EDLOS > or <6 h[36]. Agustin et al. also found that EDLOS (irrespective of whether it was >6 h) had no statistically significant impact on IHM. [39] Haji et al. did not find an association between EDLOS and IHM in patients with sepsis [51]. In their analysis, they did not use a specific EDLOS cutoff. However, Zhang et al. found that, for septic patients, EDLOS >6 h was associated with a 1.8-fold higher IHM than was EDLOS < 6 h.[44] García-Gigorro et al. observed that a prolonged EDLOS was associated with a 2.5-fold higher IHM, particularly if the EDLOS exceeded 5 h [38.] Siletz et al. found no significant difference in IHM between trauma patients with an EDLOS < 90 min and those with an EDLOS > 90 min [40]. Soni et al. also found no association between EDLOS and IHM in trauma patients [42]. Intas et al. found that ICU patients with an EDLOS of at least 6 h experienced a 5-fold higher IHM [45]. Aitavaara–Anttila et al. found that EDLOS was not associated with IHM among ICU patients [47]. Delays in exiting the ED were associated with therapeutic or diagnostic procedures. Khan et al. also found no significant mortality difference between ICU patients with an EDLOS >6 h or <6 h [48].

Groenland et al. concluded that an EDLOS exceeding 2.4 h was associated with a 1.3-fold increase in IHM in ICU patients.[50] In a prospective cohort study performed at a single-unit tertiary hospital, Santos et al. observed that prolonged EDLOS was not associated with prolonged ICU stay or increased IHM but was associated with a longer hospital stay.[52] In their study, the median EDLOS was extremely high (17 h), although they did not use a specific EDLOS cutoff. Lin et al. observed an association between delayed EDLOS and IHM [60]. An EDLOS of ≥ 6 h was associated with significant IHM, while a lower EDLOS seemed to be protective [60]. Lin et al observed that every hour of delay increased mortality [60].

Other studies that focused on the ED ICU population, and using continuous measures of EDLOS without specific cutoffs, did not find an association between increased EDLOS and IHM [53, 54, 55, 56]. Stey et al. analyzed critically injured patients directly admitted to the ICU from the ED [58]. They found no association between EDLOS and IHM, but found that women were more likely to have a prolonged EDLOS as well as higher IHM rates.[58] Choi et al. showed that prolonged EDLOS, beyond 24 h, was independently associated with all-cause IHM in older patients with infections who were admitted to the ICU [59]. Aletrebi et al. showed that EDLOS of patients admitted to the ICU was an independent risk factor for mortality [61]. The association between EDLOS and IHM was stronger when EDLOS exceeded 4 h. Rana et al. observed that longer EDLOS was associated with an increased mortality rate, particularly in patients who had high acute physiology and chronic health evaluation (APACHE) IV scores [63].

Crilly et al. reported that one in four cases had Severity Triage Scores of 3, 4, or 5, and one in four had Severity Triage Scores of 1 and 2, which represent the most critical patients [67].

Elay et al. showed that EDLOS does not affect IHM, but that patients with high disease severity scores (APACHE II score ≥20 or SOFA score ≥8) had a poor prognosis [69]. Rose et al., in a population-based study in which less than half of adult ED patients were admitted to an ICU ≤ 6 h after arrival at an ED, showed that prolonged EDLOS did not reduce 90-day mortality [3]. Sabaz et al. found that prolonged EDLOS before admission to the ICU was associated with increased mortality [70]. Verma et al. observed that longer EDLOS increased mortality, irrespective of the severity triage score at the arrival in the ED, and mostly when EDLOS exceeded 24 h [71].
