# Supplementary figures and images for: Length-of-Stay in the Emergency Department and In-Hospital Mortality: A Systematic Review and Meta-Analysis
Source: J Clin Med. 2022 Dec 21;12(1):32. doi: 10.3390/jcm12010032 (PMC9821325; doi:10.3390/jcm12010032)

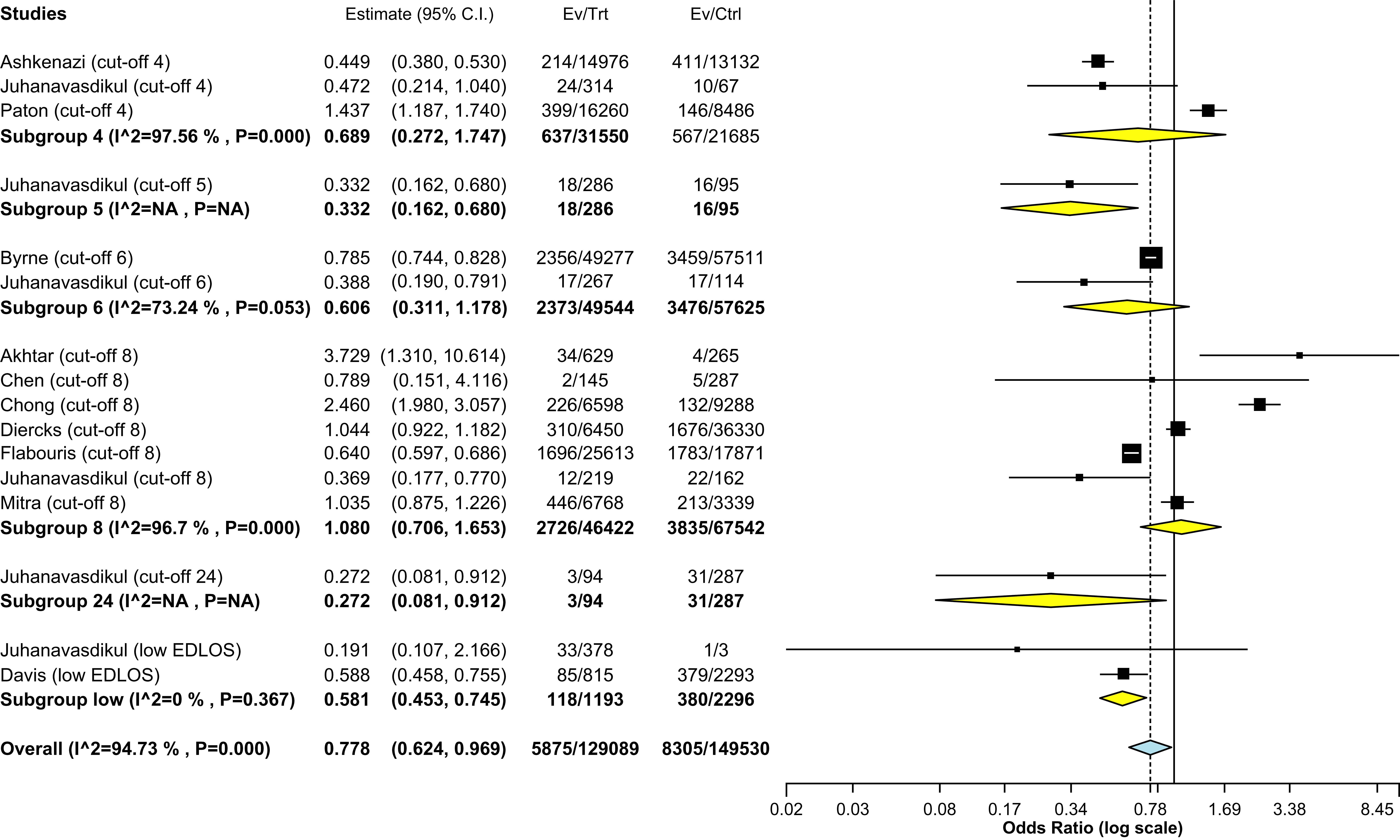

Supplement: Supplementary file 1 [file jcm-12-00032-s001.zip › Sup Fig2.tif]

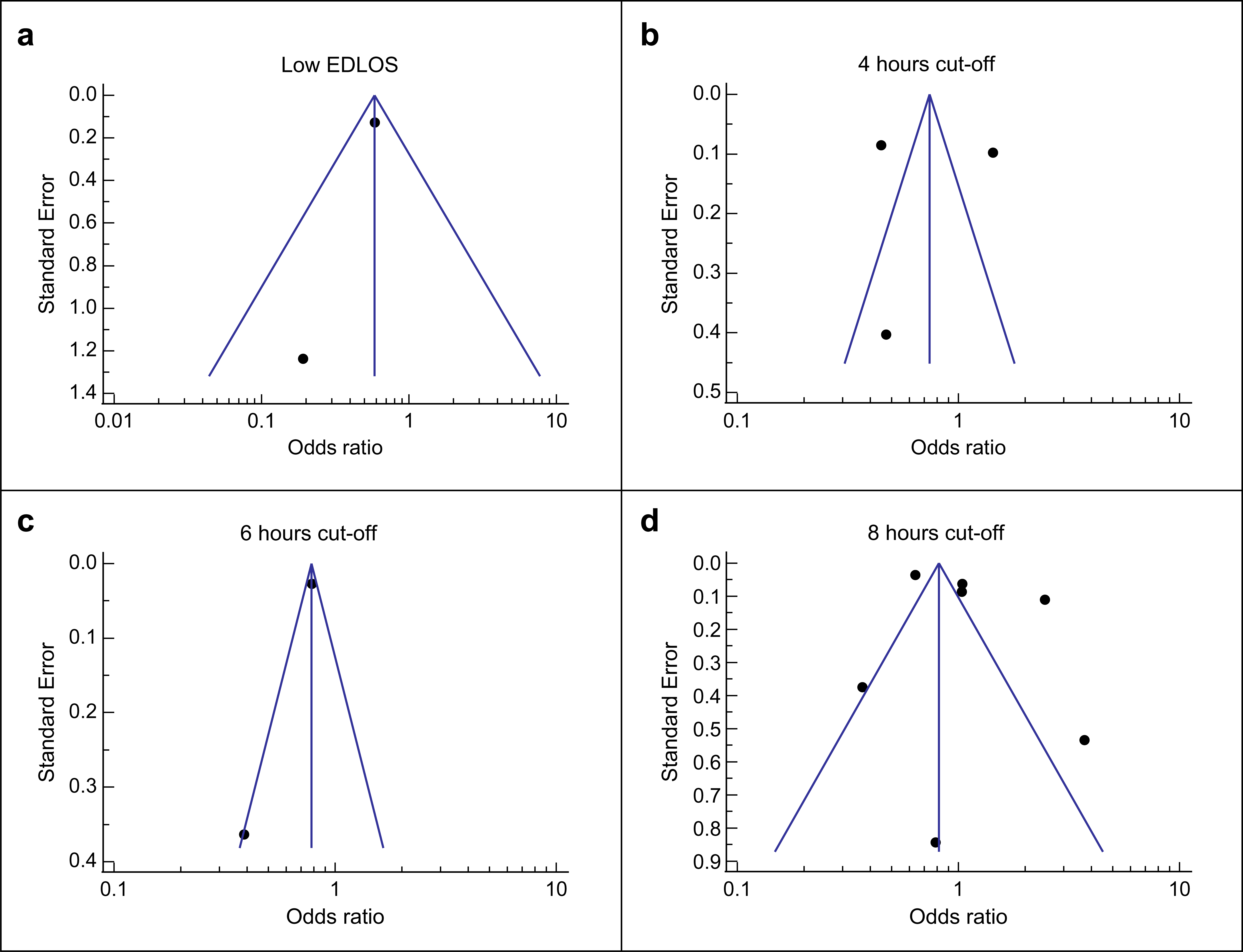

Supplement: Supplementary file 1 [file jcm-12-00032-s001.zip › Sup Fig5.tif]
